# Supplementary material for: Telomere-to-telomere Citrullus super-pangenome provides direction for watermelon breeding
Source: Nat Genet. 2024 Jul 8;56(8):1750–61. doi: 10.1038/s41588-024-01823-6 (PMC11319210; doi:10.1038/s41588-024-01823-6)

nature genetics

Supplementary information https://doi.org/10.1038/s41588-024-01823-6

Telomere-to-telomere *Citrullus* super-pangenome provides direction for watermelon breeding

In the format provided by the authors and unedited

Supplementary Note 1: T2T assemblies of 27 diverse accessions from seven species

In addition to the representative accessions of each species, we also included accessions PI 296341-FR (CA) for its Fusarium wilt resistance, PI 189225 (CA) for its anthracnose resistance, PI 482276 (CA) for its gummy stem blight resistance and PI 595203 (CM) for its resistance to multiple viruses. To investigate how the genome structure changes when introducing resistant traits from wild relatives into watermelon cultivars, we specifically chose the inbred line PKR6. This line was developed by intercrossing a watermelon cultivar with three accessions of *C. amarus* (PI 296341-FR, PI 189225, and PI 482270), along with one accession of *C. mucosospermus* (PI 595203). The development of PKR6 has introgressed several genes conferring resistance against *Fusarium oxysporum* f. sp. *niveum* race 1 and 2, anthracnose, powdery mildew, watermelon mosaic virus, and zucchini yellow mosaic virus.

Assembly quality was evaluated from several perspectives. Assembly completeness was supported by BUSCO and LAI scores, with an average BUSCO score of 99.0% and an average LAI value of 9.14 (**Table 1**). Assembly accuracy was confirmed by QV and alignment rates, with 99.5% of NGS reads, 99.96% of HiFi reads, and 99.36% of ONT reads being correctly aligned to their respective assembled genomes (**Supplementary Table 6**). An average QV value of 71.0 indicated a very low single-base error rate (**Table 1**). Finally, the genome assemblies of five species were further evaluated using BioNano optical maps, which covered almost the entire sequence of the evaluated genomes (**Supplementary Fig. 4**). Overall, the 27 assemblies matched the quality of the watermelon T2T-G42 reference genome confirming the ultra-high quality of these pangenome assemblies.

Repetitive elements in the 27 genome assemblies were annotated using Extensive *de novo* TE Annotator (EDTA). Repetitive DNA accounted for an average of 56.2% of each genome, ranging from 54.78% to 60.96% (**Table 1**). Among repetitive sequences, Gypsy was the most abundant, consistent with previous reports[1] on the watermelon genome (**Supplementary Table 7**). The observed variation in the genome sizes of *C.*

ecirrhosus and *C. rehmii* can be explained primarily on differences in the number of Transposable Elements (TEs), especially through the amplification of Long Terminal Repeats (LTRs) and DNA transposons (**Supplementary Table 7**).

To annotate protein-coding and small RNA genes, we collected various tissues at different developmental stages for each of the 27 accessions and performed RNA-seq. Using a combination of *de novo* prediction, homolog annotation, and RNA-seq assembly, we predicted an average of 24,698 protein-coding genes, 913 tRNAs, 388 snRNAs, and 1,001 rRNA genes per genome (**Supplementary Table 8**). The average gene length ranged from 3,433 bp to 3,775 bp, while the average length of coding sequences (CDS) ranged from 1,131 bp to 1,218 bp. BUSCO evaluation revealed that an average of 95.15% of the 1,614 single-copy genes were completely assembled in these genomes (**Supplementary Table 9**), indicating a high degree of gene annotation completeness. Between 86% and 93% of genes were functionally annotated by at least one of the following databases: NR, eggNOG, GO, KEGG, InterPro, and Swiss-Prot (**Supplementary Table 10**).

Supplementary Note 2: Watermelon super-pangenome construction and analysis

To establish a comprehensive gene pool for *Citrullus*, we integrated the publicly available G42 and *C. naudinianus* female genomes into our pangenome analysis (https://www.dnazoo.org/post/citrullus-naudinianus-sond-hook-f). Orthofinder2 was used to cluster 686,583 predicted gene models from 28 accessions into non-redundant 32,513 pan-gene families, forming a pangenome based on protein-coding genes.

To further characterize the watermelon pangenome, we categorized gene families into four groups based on their frequency of occurrence. Gene families present in all species or genomes are defined as core genes, those present in 26-27 genomes as soft-core genes, those present in 2-6 species or 2-25 genomes as dispensable genes, and those only present in one species or genome as private genes. Functional enrichment analysis

based on protein domains shows that core genes are enriched in domains involved in a broad range of plant growth and developmental functions. These include ring finger domains, AP2 domains and WD domains (**Supplementary Fig. 6e-f**). In contrast, domains associated with retrotransposons are enriched in dispensable genes (**Supplementary Fig. 6g,h**). The private genes of *C. naudinianus* and *C. colocynthis* are enriched for disease resistance-related domains, while the private genes of *C. lanatus* are enriched for carbohydrate binding or metabolism-related domains (**Supplementary Fig. 6i,j**).

Supplementary Note 3: SV characterization and graph-based genome

To extend our understanding of SVs from the 28 accessions to a population scale, we performed population SV and PAV identification. A total of 18,985 SVs and 157,060 PAVs greater than 50 bp were identified from 402 watermelon populations[1]. By integrating SVs identified in the super-pangenome, population SVs, population SNP variations, and the G42 reference genome sequence, a graph-based pangenome was constructed. This graph-based super-pangenome serves as a valuable platform for genotyping SVs in populations and for performing SV-based association analyses for important agronomic traits of watermelon. To facilitate access to these resources, a web-based database (**www.watermelondb.cn**) was developed. This tool integrates a Genome Browser and BLAST functionality, enabling researchers to carry out various analyses using high-quality assembly sequences. It also allows for the rapid determination of expression levels, variation information, and the metabolome landscape during fruit development for any gene of interest.

Supplementary Note 4: Gene gain and loss during watermelon domestication

Our study also discovered variations in nucleotide binding sites and leucine-rich repeats

(NLRs), which encode plant resistance genes[2], with *C. naudinianus* housing the highest number of NLRs (53), while *C. rehmii* possessed the lowest count (31), and other species ranging from 40 to 46 (**Supplementary Table 21**). Additionally, the LOX gene family associated with pathogen invasion resistance[3], exhibited expansion in *C. naudinianus* compared to other species. The *C. naudinianus* genome contained significantly more LOX genes (51), with most of them located on Chr11 (**Fig. 5b**). Other species had fewer LOX genes located on Chr02. The partial loss of LOX genes in *C. naudinianus* may be related to a chromosomal rearrangement event involving Chr11 and Chr02.

Furthermore, the loss of gene clusters associated with disease resistance functions during the evolution of cultivated watermelon was observed, including CRISPs[4], R[5], LRR[6], and PR[7] gene families (**Supplementary Fig. 12**). These 13 gene clusters were widely distributed in the watermelon CWR and were classified into different cluster families based on their functional domains (**Supplementary Table 22**). To address this, efforts to develop new germplasm incorporating lost resistance genes from *C. colocynthis* and *C. amarus*, through intercrossing with *C. lanatus*, are vital for watermelon breeding programs.

Supplementary Note 5: Cucurbitacin and sugar gene networks in watermelon species

Key metabolite differences between different watermelon CWRs are striking. To reveal the influence of key genes on flavor formation during watermelon development, we assessed the dynamic changes of major metabolites using LC-MS analysis. We selected 11 representative watermelon fruit samples from five species, harvested at 10, 18, 26, 34, and 42 days after pollination (DAP). Fruits of different species show distinct variations in fruit size, flesh color, and flesh firmness (**Supplementary Fig. 14c,d**). PCA analysis of transcriptomic data classified the watermelon samples into four

distinct groups, while PCA based on metabolomics data divided them into five groups (**Supplementary Fig. 14a,b**). Integrating PCA results from both omics in fruit revealed significant genetic differences among *Citrullus* species. Fruits at early developmental stages (A and B) tended to form a separate branch.

Based on the abundance of metabolites captured in the samples, 3077 detected metabolites (**Supplementary Table 24**) were clustered into eight modules (**Supplementary Fig. 15a**). ME3 represents substances accumulated during the development of the cultivated watermelon. Saccharolipids and monosaccharides are enriched in ME3. ME5 denotes metabolites that accumulated in *C. mucosospermus*, with lignans and neolignanes being enriched in this module. ME4 refers to metabolites that accumulated in *C. rehmii* over time, with isoflavonoids being enriched in this module. ME2 and ME8 represent metabolites that accumulated in *C. amarus*, with flavonoids and triterpenoids being enriched in this species. ME1 and ME7 denote metabolites that accumulate in *C. colocynthis*. All 11 cucurbitacin substances that we captured are enriched in ME1 (**Supplementary Fig. 15b**).

Previous research has shown that the main form of soluble sugar in watermelon fruit is sucrose, which determines sweetness[8]. In total, 901 genes involved in sugar metabolism were identified that were highly correlated (average r=0.74) with sucrose content during fruit development (**Supplementary Table 25**). These included *ClAGA2* (*ClG42_04g0035700*)[8], *ClNAC68* (*ClG42_03g0079100*)[9], *ClSWEET3* (*ClG42_01g0006000*)[10], *ClVST1* (*ClG42_02g0044600*)[11] and *TST2*[12]. All of these have been shown to be involved in sugar accumulation and metabolism during fruit maturation. The expression patterns of these genes align with fruit sucrose content at different developmental stages. We linked several genes encoding various metabolic enzymes, such as glycosyltransferase, SWEET, and pectinesterase (**Supplementary Fig. 15c**) with fruit quality traits. In total, 40 key genes were found to be significantly positively correlated with sucrose content, suggesting that they play various roles in increasing sugar transport and accumulation. These genes may be potential candidates

for manipulating watermelon flavour.

Cucurbitacin is the key metabolite determining the bitterness of watermelon. The accumulation of cucurbitacin has been significantly reduced and even eliminated during watermelon domestication. The main component of cucurbitacins in watermelon is cucurbitacin E (CuE). To identify potential genes closely related to cucurbitacin metabolism, we applied WGCNA to correlate differentially expressed genes with cucurbitacins P, I, F, E, and D. Cucurbitacin biosynthesis primarily involves the formation of terpenoid skeletons and oxidative modifications, with modification reactions usually controlled by acetyltransferases (ACT), cytochrome P450 (CYP450), and oxidosqualene cyclases (OSC). We observed that the expression of numerous candidate genes from MATE (average r=0.76), ERF (average r=0.82), DHO (average r=0.84), CYP450 (average r=0.85), ACT (average r=0.84), and bHLH (average r=0.82) gene families closely correlated with cucurbitacin biosynthesis (**Supplementary Fig. 15d** and **Supplementary Table 26**). Their expression patterns aligned with the cucurbitacin content (were highly expressed in *C. colocynthis* and not expressed in other species) at different developmental stages.

To further validate the candidate genes related to sugar and cucurbitacin accumulation, we examined whether the DNA sequences of these genes show variation between species. We found that insertion/deletions (InDels) of more than 5 bp occurred primarily in *C. colocynthis* and *C. amarus*, while they were not observed in *C. mucosospermus* and *C. lanatus*. In addition, most InDels were located 1kb upstream of those genes (**Supplementary Table 27**). The expression profiles of 21 key candidate genes identified from the co-expression network, across five stages of fruit development, were examined using RT-qPCR. RT-qPCR results confirmed that the expression patterns of these 21 genes were consistent with the transcriptome data (**Supplementary Fig. 13a,b**).

Supplementary Note 6: Trait-related candidate genes in the selective sweeps

To explore novel candidate genes harboring SVs that may contribute to trait diversity, selective sweeps were made to compare wild and cultivated watermelons. In total, 1,750 genes from selective sweeps were detected by the selective sweeps and 79 of them had SVs in their coding regions (**Supplementary Table 28**). Using the Swiss-Prot database and the analysis of transcriptomic data, candidate genes potentially associated with other important functions were identified (**Supplementary Table 29**). *ClG42_02g0065900* belongs to the leucine-rich repeat receptor-like protein kinase family which has been reported to be associated with immune responses, activation of cell death, and plant development processes[13]. A 30-bp insertion was detected in the first exon of *ClG42_02g0065900* in *C. colocynthis* and a 24-bp deletion in *C. amarus* (**Fig. 6**). *ClG42_09g0186900*[14] was predicted to encode a transcription factor MYB106 involved in the activation of cuticular wax biosynthesis under drought stress. Homolog of another gene, *ClG42_03g0052200*, was reported to associated with gain and loss of fruit flavor compounds in strawberry[15]. The homologous genes of *ClG42_06g0011700*, *ClG42_06g0112900* and *ClG42_08g0009100* were reported to be associated with the growth and development of rice and *Arabidopsis*[16-18]. Accelerating the functional analysis of these candidate genes will facilitate their exploitation and utilization of these and other functional genes.

Supplementary references

1. Guo, S., et al. Resequencing of 414 cultivated and wild watermelon accessions identifies selection for fruit quality traits. *Nat. Genet.* **51**, 1616–1623 (2019).
2. Sun, H., et al. Karyotype stability and unbiased fractionation in the paleo-allotetraploid cucurbita genomes. *Mol. Plant* **10**, 1293–1306 (2017).
3. Viswanath, K.K., et al. Plant lipoxygenases and their role in plant physiology. *J. Plant Biol.* **63**, 83–95 (2020).
4. Gibbs, G.M., Roelants, K. & O'Bryan, M.K. The CAP superfamily: cysteine-rich secretory proteins, antigen 5, and pathogenesis-related 1 proteins-roles in reproduction, cancer, and immune defense. *Endocr. Rev.* **29**, 865–897 (2008).
5. Jung, H.W., et al. Pathogen-associated molecular pattern-triggered immunity involves proteolytic degradation of core nonsense-mediated mRNA decay factors during the early defense response. *Plant Cell* **32**, 1081–1101 (2020).
6. Wang, J., et al. Ligand-triggered allosteric ADP release primes a plant NLR complex. *Science* **364**, eaav5868 (2019).
7. Zribi, I., Ghorbel, M. & Brini, F. Pathogenesis related proteins (PRs): from cellular mechanisms to plant defense. *Curr. Protein Pept. Sci.* **22**, 396-412 (2021).
8. Ren, Y., et al. Evolutionary gain of oligosaccharide hydrolysis and sugar transport enhanced carbohydrate partitioning in sweet watermelon fruits. *Plant Cell* **33**, 1554–1573 (2021).
9. Wang, J., et al. The NAC transcription factor ClNAC68 positively regulates sugar content and seed development in watermelon by repressing ClINV and ClGH3.6. *Hortic. Res.* **8**, 214 (2021).
10. Gong, C., et al. Multi-omics integration to explore the molecular insight into the volatile organic compounds in watermelon. *Food Res. Int.* **166**, 112603 (2023).
11. Ren, Y., et al. Localization shift of a sugar transporter contributes to phloem unloading in sweet watermelons. *New Phytol.* **227**, 1858–1871 (2020).
12. Deng, Y., et al. A telomere-to-telomere gap-free reference genome of watermelon and its mutation library provide important resources for gene discovery and breeding. *Mol. Plant* **15**, 1268–1284 (2022).
13. Soltabayeva, A., et al. Receptor-like kinases (LRR-RLKs) in response of plants to biotic and abiotic stresses. *Plants* **11**, 2660 (2022).
14. Lee, S.B., Kim, H.U. & Suh, M.C. MYB94 and MYB96 additively activate cuticular wax biosynthesis in *Arabidopsis*. *Plant Cell Physiol.* **57**, 2300–2311 (2016).
15. Aharoni, A., et al. Gain and loss of fruit flavor compounds produced by wild and cultivated strawberry species. *Plant Cell* **16**, 3110–3131 (2004).
16. Sugiyama, Y., Wakazaki, M., Toyooka, K., Fukuda, H. & Oda, Y. A novel plasma membrane-anchored protein regulates xylem cell-wall deposition through microtubule-dependent lateral inhibition of Rho GTPase domains. *Curr. Biol.* **27**, 2522–2528.e4 (2017).
17. Yan, Y., et al. A MYB-domain protein efm mediates flowering responses to environmental cues in *Arabidopsis*. *Dev. Cell* **30**, 437–448 (2014).

18. Nonogaki, H., Gee, O.H. & Bradford, K.J. A germination-specific endo-β-mannanase gene is expressed in the micropylar endosperm cap of tomato seeds, *Plant Physiol.* **4**, 1235–1246 (2000).
19. Xuan, C., et al. Systematic Genome-wide study and expression analysis of sweet gene family: sugar transporter family contributes to biotic and abiotic stimuli in watermelon. Int J Mol Sci. 22, 8407 (2021).
20. Hong, J., Hossain, M., Jung, H. & Nou, I. QTL associated with Gummy Stem Blight (GSB) resistance in watermelon. BMC Genomics. 23, 632 (2022).
21. Zhang, M., et al. CRISPR/Cas9-mediated mutagenesis of Clpsk1 in watermelon to confer resistance to Fusarium oxysporum f.sp. niveum. Plant Cell Rep. 39, 589-595 (2020).
22. Prothro, J., et al. QTL associated with sex expression in an inter-subspecific watermelon population. JASHS. 138, 125-130 (2013).
23. Gong, C., et al. An integrated transcriptome and metabolome approach reveals the accumulation of taste-related metabolites and gene regulatory networks during watermelon fruit development. Planta. 254, 35 (2021).
24. Guo, S., et al. Comparative transcriptome analysis of cultivated and wild watermelon during fruit development. *PLoS ONE* **10**, e0130267 (2015).
25. Manzano, S., et al. The ethylene biosynthesis gene citacs4 regulates monoecy/andromonoecy in watermelon (*Citrullus lanatus*). *PLoS ONE*, **11**, e0154362 (2016).
26. Fang, X. et al. Expression of *ClPAP* and *ClPSY1* in watermelon correlates with chromoplast differentiation, carotenoid accumulation, and flesh color formation. *Sci. Hortic.* **270**, 109437 (2020).
27. Jiang, H., et al. RNA-seq analysis of watermelon (*Citrullus lanatus*) to identify genes involved in fruit cracking. *Sci. Hortic.* **248**, 248–259 (2019).
28. Yuan, P., et al. Watermelon domestication was shaped by stepwise selection and regulation of the metabolome. *Sci. China Life Sci.* **66**, 579–594 (2023).
29. McGregor, C., Waters, V., Vashisth, T. & Abdel-Haleem, H. Flowering time in watermelon is associated with a major quantitative trait locus on chromosome 3. *J Am Soc Hortic Sci.* **139**, 48–53 (2014).
30. Shang, H., et al. Genome-wide identification and expression of the pin auxin efflux carrier gene family in watermelon (*Citrullus lanatus*). *Agriculture*, **11**, 447 (2021).
31. Umer, M., et al. Expression pattern of sugars and organic acids regulatory genes during watermelon fruit development. *Sci. Hortic.* **265**,109102 (2020).
32. Zhou, Y., et al. Convergence and divergence of bitterness biosynthesis and regulation in Cucurbitaceae. *Nat. Plants* **2**, 16183 (2016).
33. Anees, M., et al. Identification of key gene networks associated with cell wall components leading to flesh firmness in watermelon. *Front. Plant Sci*. **12**, 630243 (2021).
34. Zhang, R., et al. Disruption of the bHLH transcription factor Abnormal Tapetum 1 causes male sterility in watermelon. *Hortic Res.* **8,** 258 (2021).

35. Gimode, W., et al. (2020). QTL associated with gummy stem blight resistance in watermelon. *Theor Appl Genet.* **134**, 573-584 (2021).
36. Anees, M., et al. Aux/IAA gene *Cla004102*, is involved in synergistic regulation of various endogenous hormones, regulating flesh firmness in watermelon. *Sci. Hortic.* **310**, 111719 (2023).
37. Zhong, Y., et al. Root-secreted bitter triterpene modulates the rhizosphere microbiota to improve plant fitness. *Nat. Plants* **8**, 887–896 (2022).
38. Zhang, J., et al. High-level expression of a novel chromoplast phosphate transporter ClPHT4;2 is required for flesh color development in watermelon. *New Phytol.* **213**, 1208–1221 (2017).
39. Liu, S., et al. Nucleotide variation in the phytoene synthase (*ClPsy1*) gene contributes to golden flesh in watermelon (*Citrullus lanatus* L.). *Theor. Appl. Genet.* **135**, 185–200 (2022).
40. Dou, J., et al. Genome-wide analysis of IQD proteins and ectopic expression of watermelon ClIQD24 in tomato suggests its important role in regulating fruit shape. *Front. Genet.* **13**, 993218 (2022).
41. Wang, Y., et al. CRISPR/Cas9-mediated mutagenesis of ClBG1 decreased seed size and promoted seed germination in watermelon. *Hortic. Res.* **8**, 70 (2021).
42. Li, N., et al. A 13.96-kb chromosomal deletion of two genes is responsible for the tomato seed size in watermelon (*Citrullus lanatus*). *Plant Breed.* **140**, 944-952 (2021).

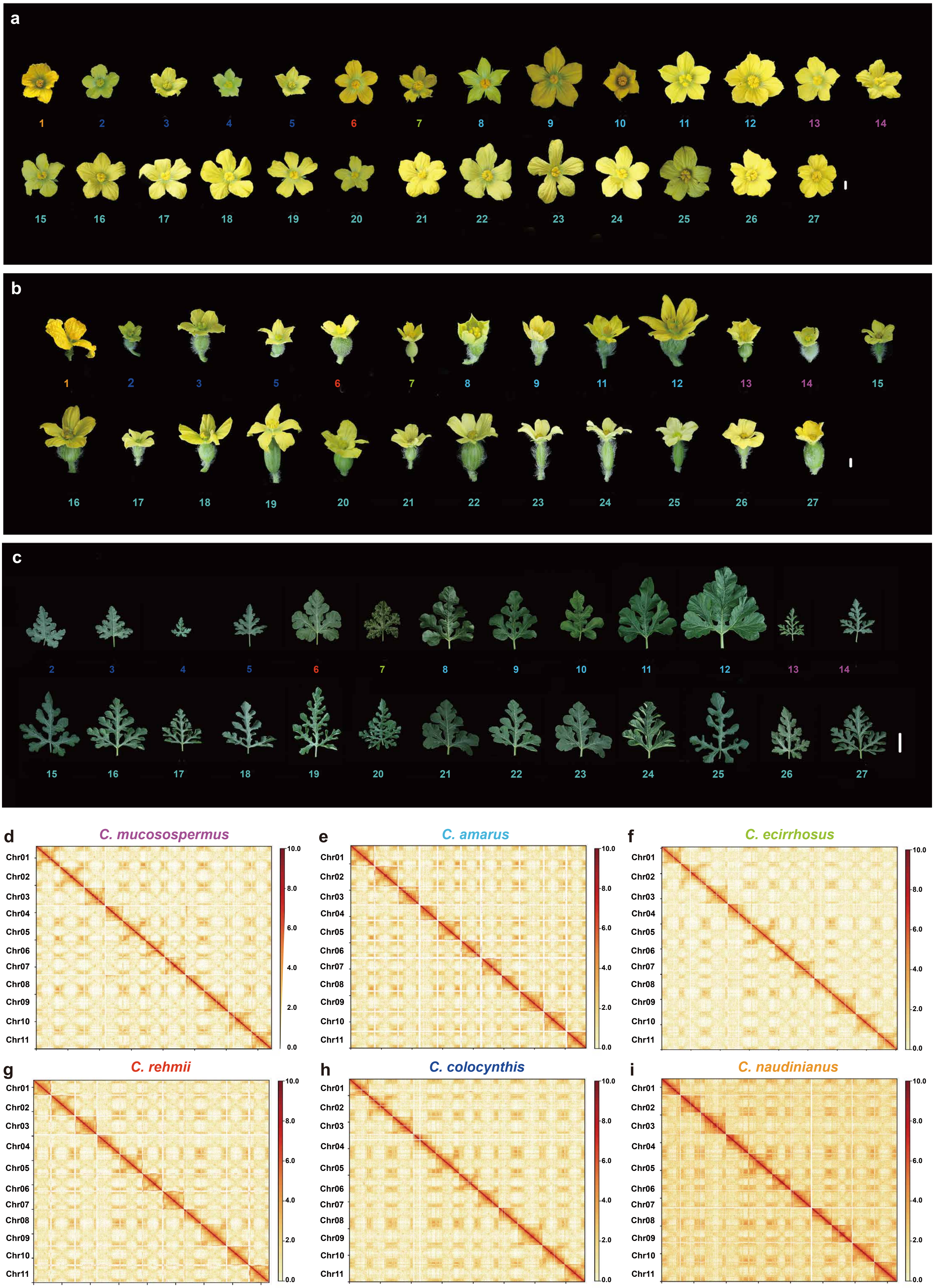

Supplementary Fig. 1 Phenotypes of selected watermelon accessions and Hi-C interaction heatmaps of six species. (a–c) Diverse plant organ phenotypes of watermelon accessions used in this study, including female flower (a), male flower (b), and leaf (c). Scale bar corresponds to 1 cm for a and b, 5cm for c. Information of accessions represented by numbers at the bottom is shown on Table S1. (d-i) The chromatin interaction map from Hi-C for the six species assembly was computed at 500 kb resolution. The heatmap illustrates the frequency of chromatin interactions across the genome and the color scale from shallow to deep indicates the interaction intensity.

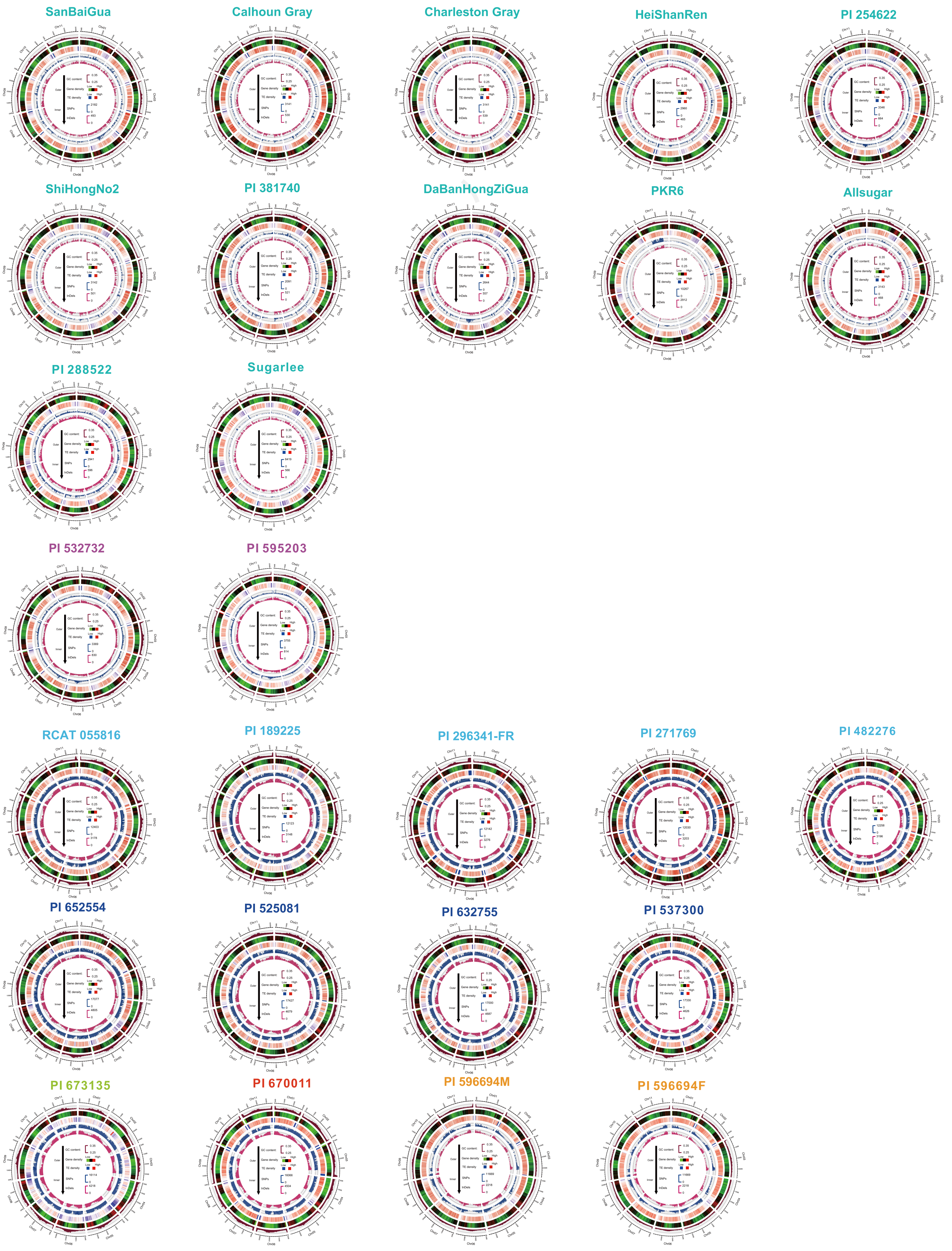

Supplementary Fig. 2 Genomic landscape Circos plot of watermelon accessions.
The distribution of genomic elements and variations were calculated across the assembled genomesof 27 accessions. Gene and transposable element (TE) densitieswere assessed in 500 kb intervals.

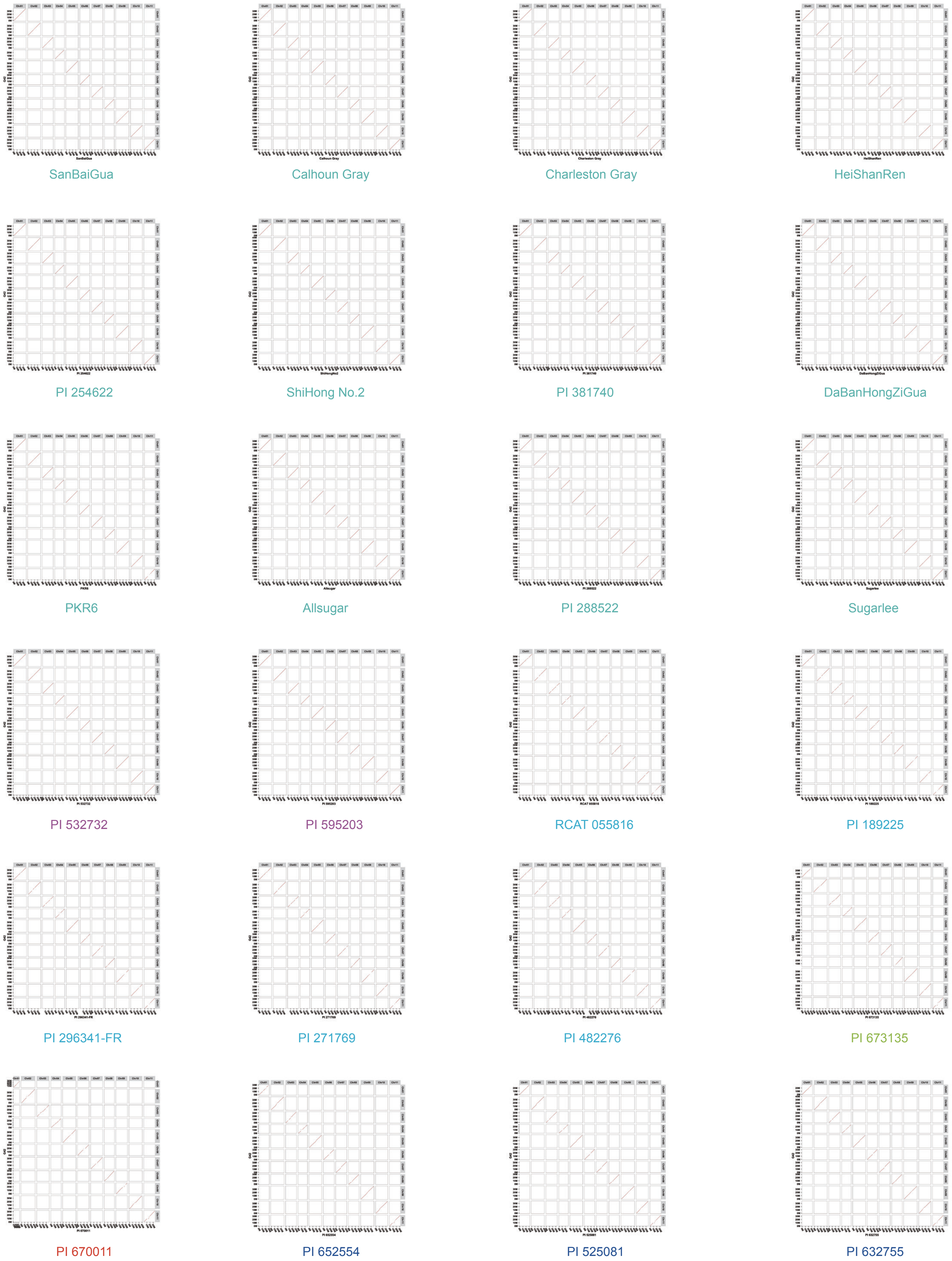

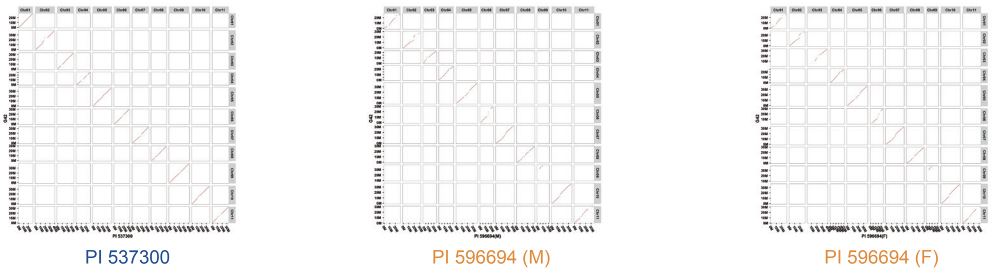

Supplementary Fig. 3 Genomic collinearity of 27 watermelon accessions genomes with the reference genome G42.
Dotplot illustrating genomic alignment between the genomes of 27 accessions and G42 genomes.

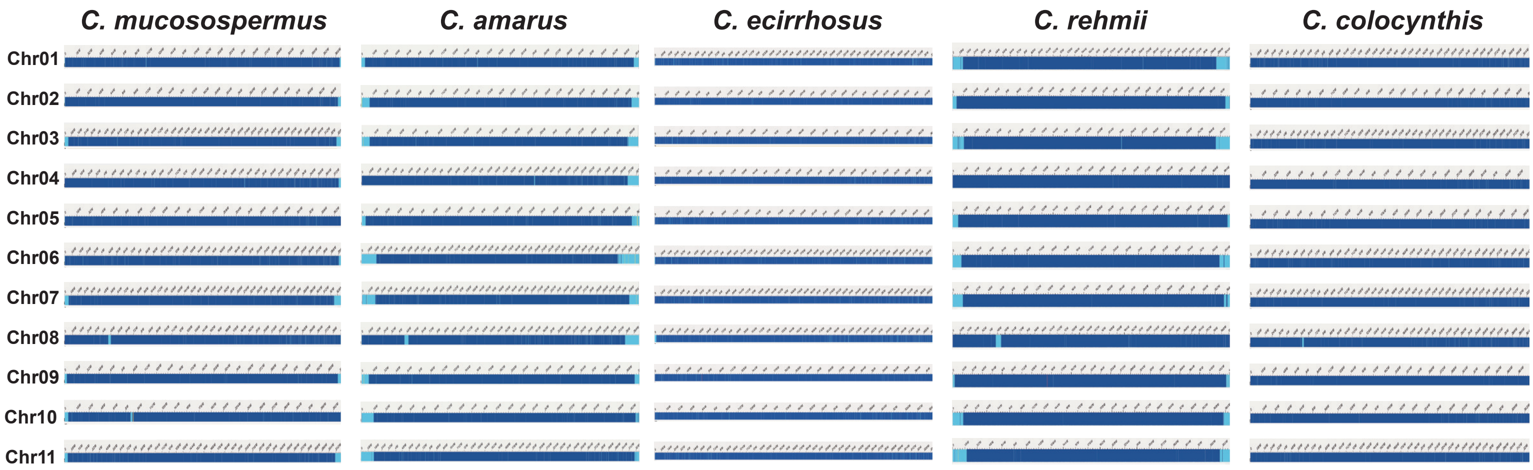

Supplementary Fig. 4 Assembly quality assessment using Bionano maps and PCR validations.
Bionano analysis of the genome assembly. The blue labels are the aligned labels, and the light blue labels are the no enzyme sites region, whereas the yellow ones are unaligned labels. The panel shows Bionano optical mapping data in 11 chromosomes have good quality with no yellow labels.

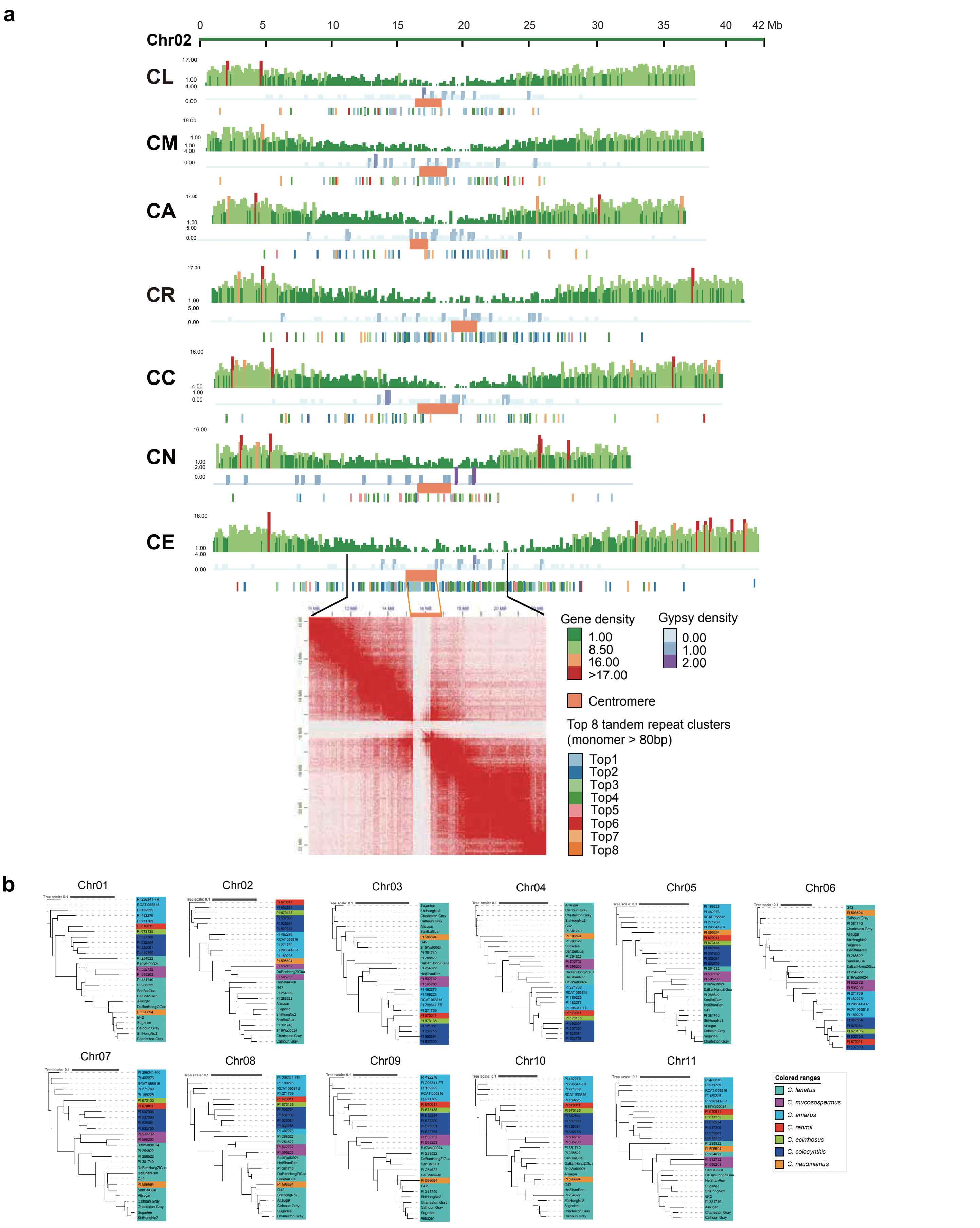

Supplementary Fig. 5 Characterization of watermelon centromere.
(a) Characterization of the centromere on Chr02 of seven species. Orange range represents the candidate centromere region. Gene densities, Gypsy LTR number and tandem repeats (with monomer length greater than 80 bp) are illustrated along the chromosome. Genome-wide Hi-C interaction heatmaps show 3D genome architecture of CE Chr02. The seven species are: CA - *C. amarus* (RCAT 055816), CC - *C. colocynthis* (PI 632755), CE - *C. ecirrhosus* (PI 673135), CM - *C. mucosospermus* (PI 595203), CN - *C. naudinianus* (PI 596694), CR - *C. rehmii* (PI 670011), CL - *C. lanatus* (PKR6). (b) The phylogenetic tree of centromeric sequences on each chromosome among the 27 genomes.

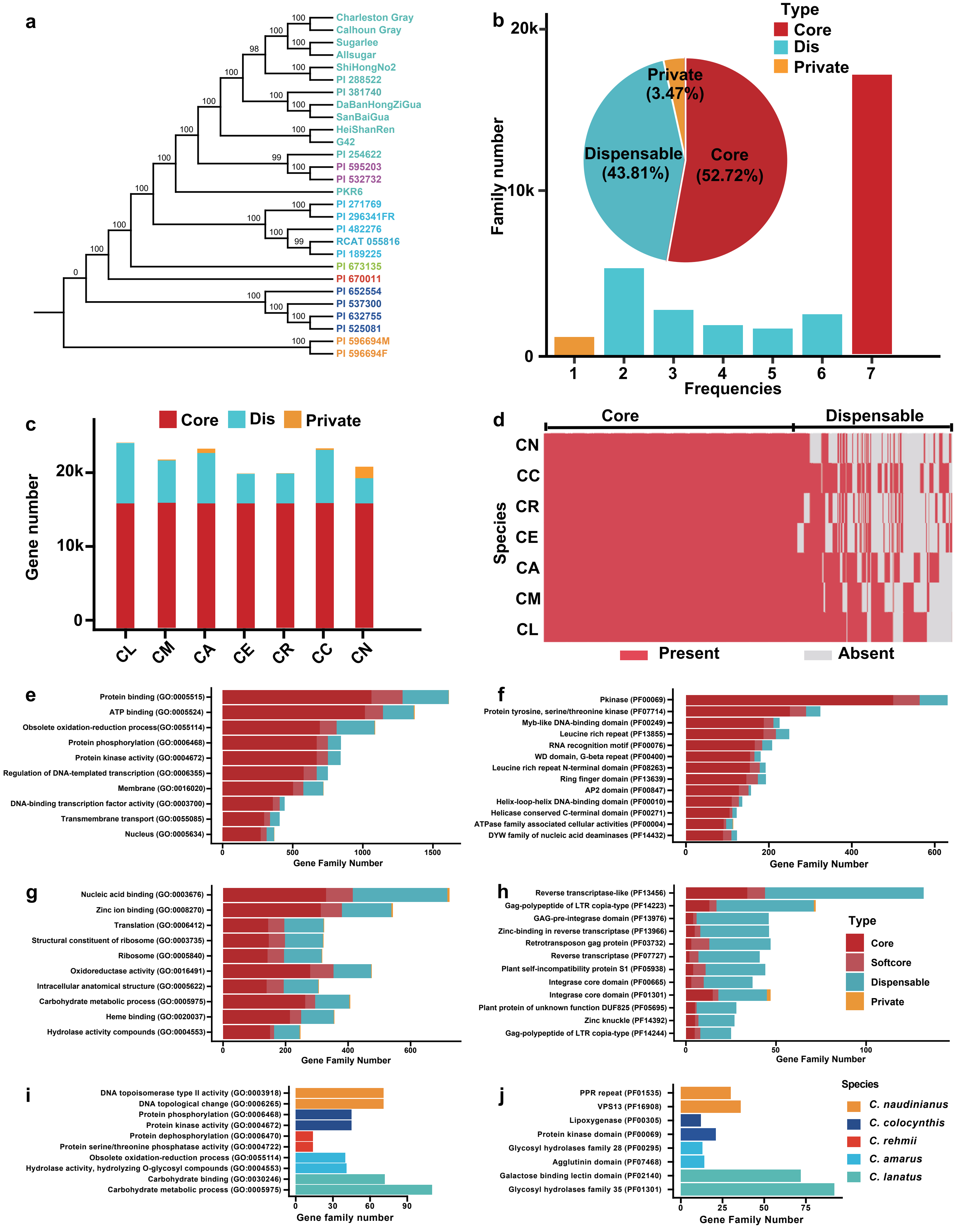

Supplementary Fig. 6 Pangenome analysis of seven watermelon species.
(a) Maximum likelihood tree based on 12,014 single-copy ortholog genes. (b) Compositions of core, dispensable, private genes of the species-level pangenomes. (c) Gene numbers of each gene type in seven watermelon species. (d) Mapping the landscape of presence-absence across non-redundant gene families for seven watermelon species. (e-j) Gene ontology enrichments of core (e) and dispensable (g) gene families. Pfam domain enrichments of core (f) and dispensable (h) gene families. Gene ontology enrichments of species private gene families (i). Pfam domain enrichments of species private gene families (j).

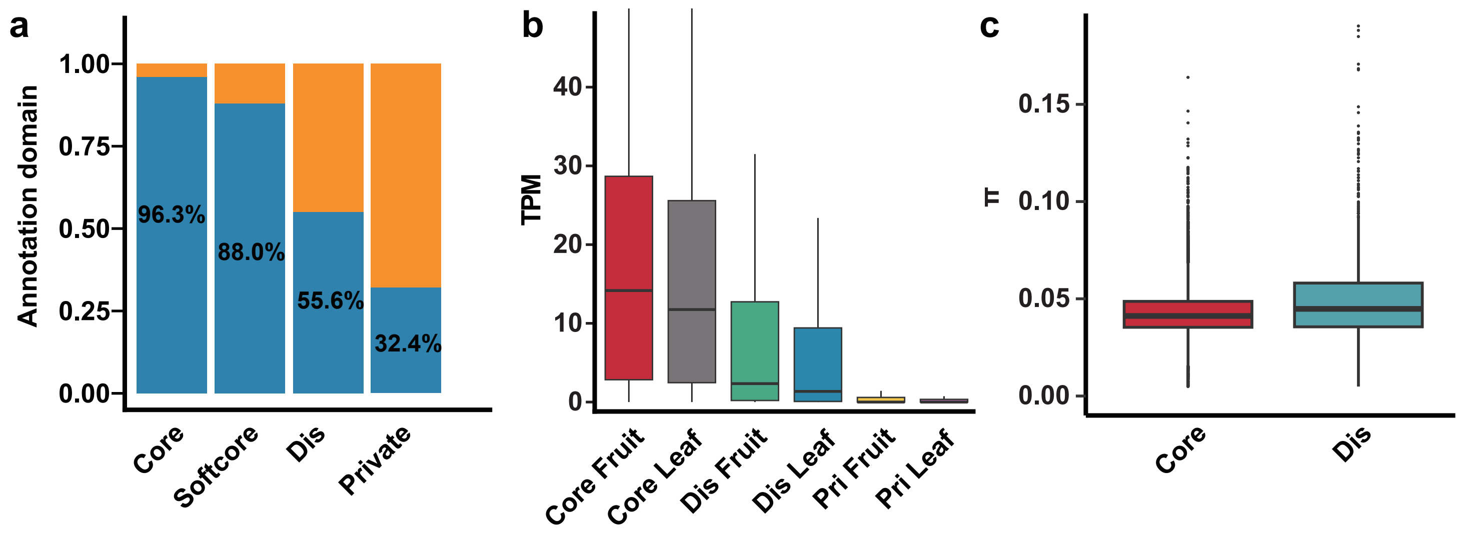

Supplementary Fig. 7 Characteristics of the watermelon pan-genome

(a) Percentage of InterPro protein domain annotations across core, soft-core, dispensable, and private gene subsets. (b) Gene expression (TPM) patterns profile in fruit and leaf tissues among core (18,117), dispensable (5,413) and private (283) gene families. (c) Compositions of nucleotide diversity (π) in core (18,117) and dispensable (5,413) genes. The edges and the centerlines of the boxes represent the IQR and medians, with the whiskers extending to the most extreme points (1.5× IQR).

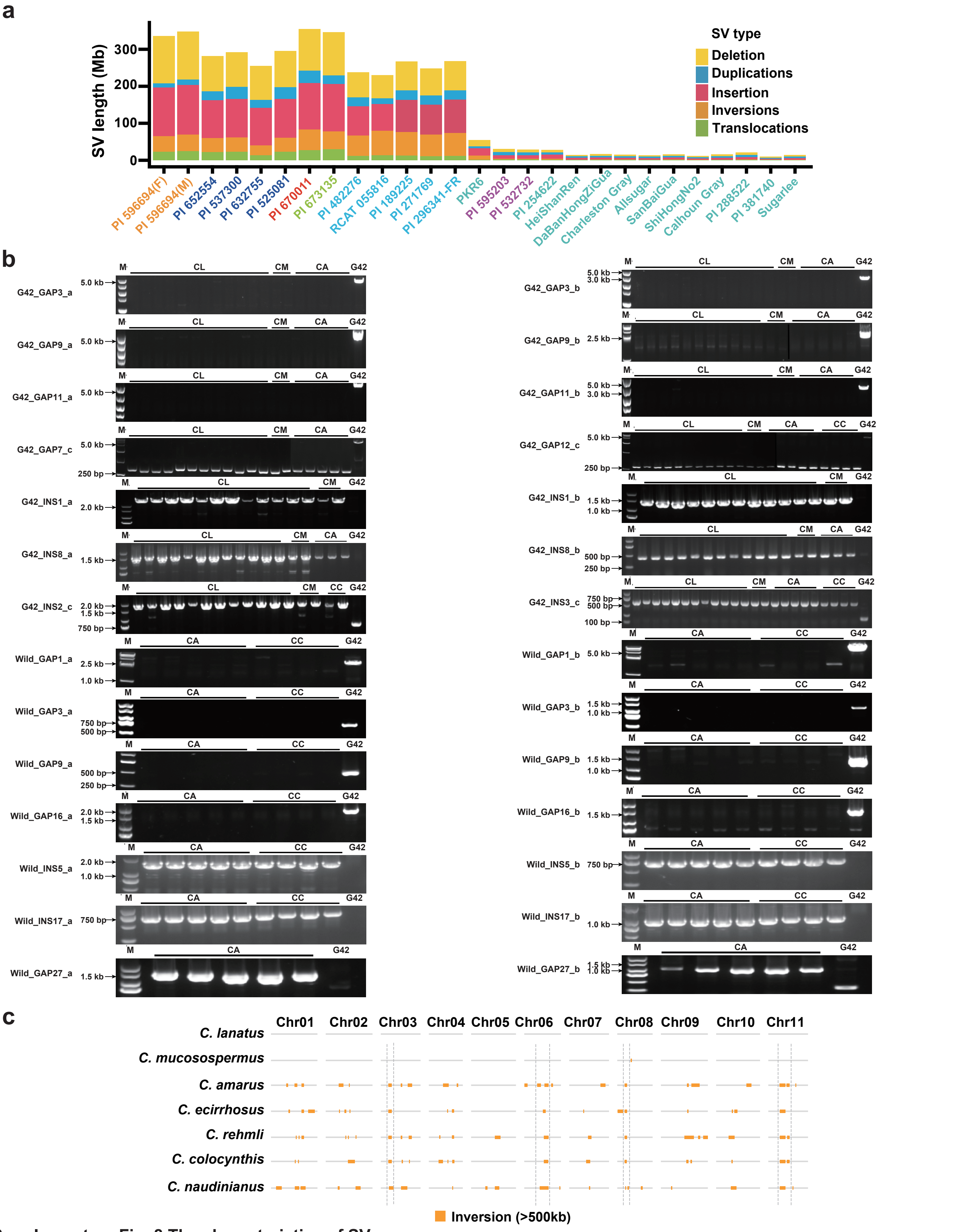

Supplementary Fig. 8 The characteristics of SVs

(a) The bar graph displays the cumulative length of the SVs, corresponding to the SVs indicated in Fig3a. (b) PCR validation of detected insertions and deletions between G42 and other watermelon accessions. Primers were designed in G42 genome to validate gaps; amplification can only be detected in G42. For insertion validation, primers were designed in inserted sequence region, no amplification from G42. For large GAPs and insertions, two pairs of primers were designed around both ends (a for 5' end primer pair, b for 3' end primer pair). CL - *C. lanatus*, CM - *C. mucosospermus*, CA - *C. amarus*, CC - *C. colocynthis*. PCR validation of detected insertions and deletions was repeated at least two times. (c) Pan-genome-based map of large inversions. The orange rectangles denote greater than 500Kb inversions.

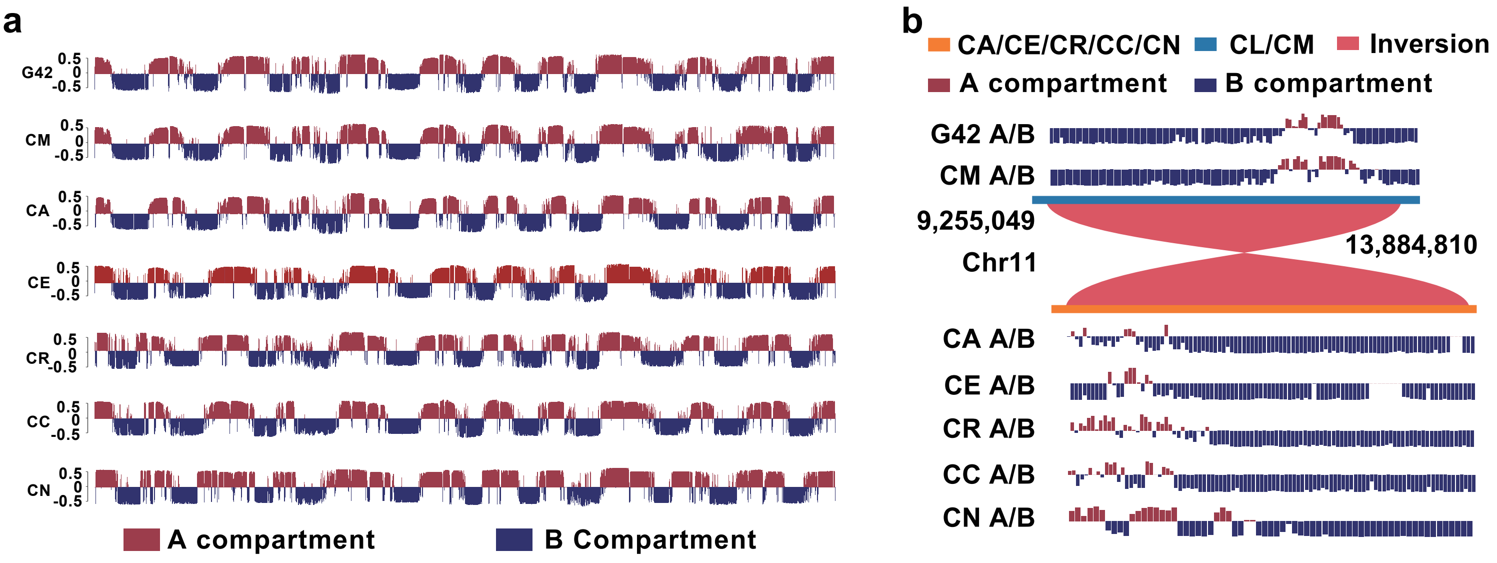

Supplementary Fig. 9 A/B compartments prediction of seven watermelon species.
(a) The A and B compartments were delineated utilizing the first principal component (PC1) extracted from the Pearson Correlation matrix analysis. (b) Switching of A/B compartment status due to a large Inversion (Red).

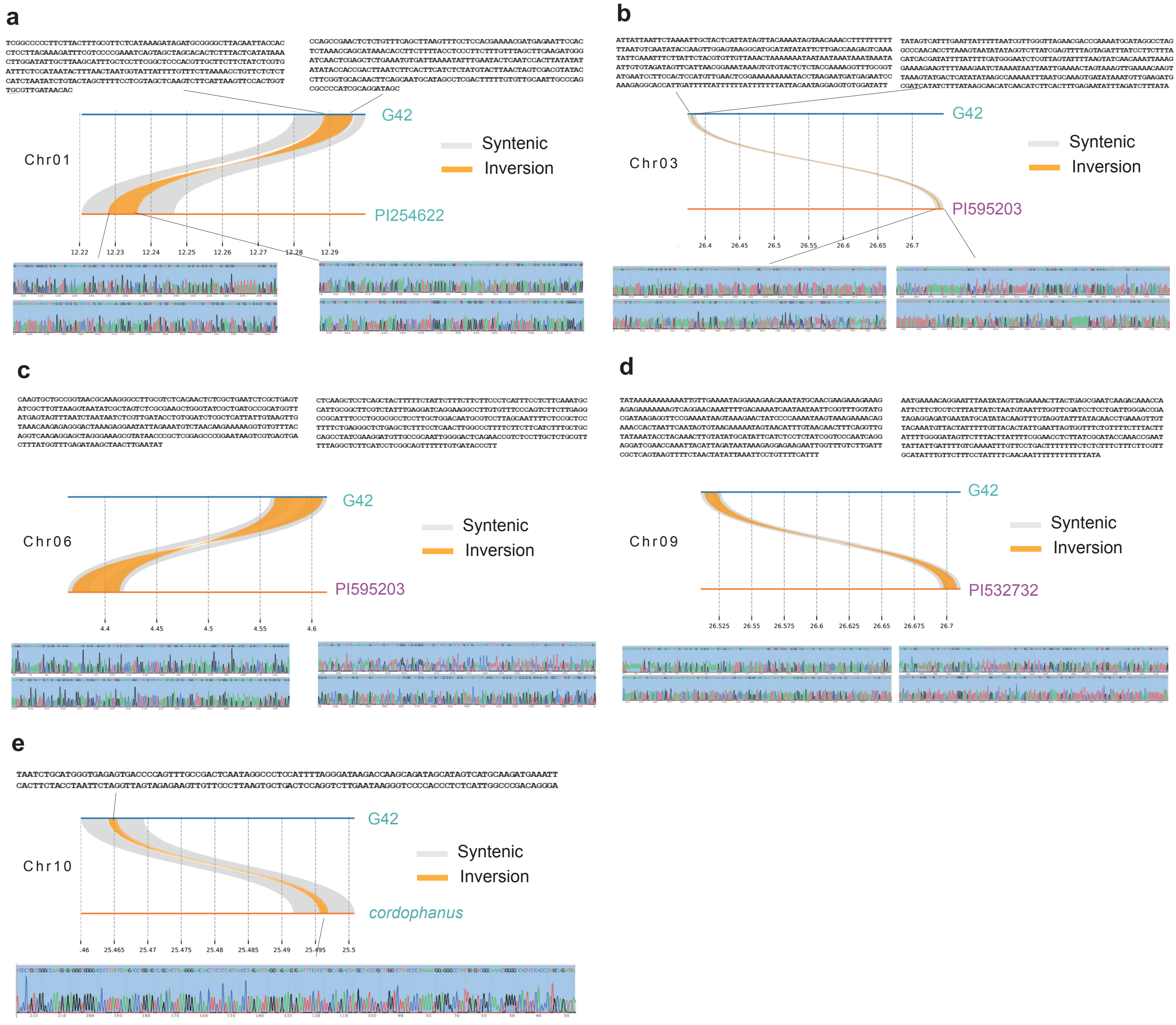

Supplementary Fig. 10 Evidence illustrating ancestor of watermelon.
(a-e) Sanger sequencing validation of inversions listed in Table S20. (a) for INV548 and INV757. (b) for INV481, INV900, and INV910. (c) for INV921. (d) for INV678 and INV933. (e) for INV397, INV484, INV912, and INV919.

		ClG42_02g0084300	ClG42_03g0058200	ClG42_03g0152400	ClG42_07g0073000
**Nucleotide divergence**		G / A	G / T	C / A	G / A
C. mucosospermus	PI 595203	G	G	C	G
	PI 532732	G	G	C	G
C. lanatus subsp. *cordophanus*	PI 254622	A	T	A	A
C. lanatus landrace	Dabanhongzigua	A	T	C	G
	Sanbaigua	A	T	C	G
	Heishanren	A	G	C	G
	PI 381740	A	G	C	G
	PI 288522	A	G	C	G
C. lanatus cultivar	Sugarlee	G	T	C	G
	Calhoun Gray	G	T	C	G
	Allsugar	G	T	C	G
	Shihong No.2	G	G	C	G
	Charleston Gray	G	T	C	G
	PKR6	G	G	C	G

Supplementary Fig. 11 SNP detected in coding sequence region of four genes among accessions from *C. mucosospermus*, *C. lanatus* subsp. *cordophanus*, and *C. lanatus*.

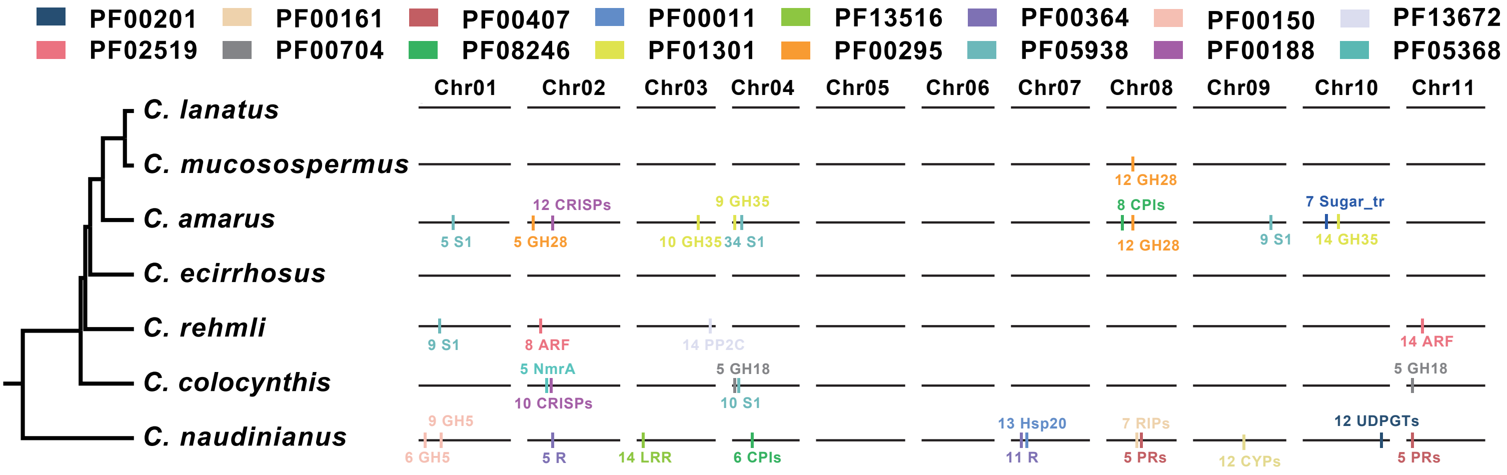

Supplementary Fig. 12 The distribution of gene clusters lost on the chromosomes of seven watermelon species. Gene clusters lost during the evolutionary of cultivated watermelon are extensively distributed in wild watermelon.

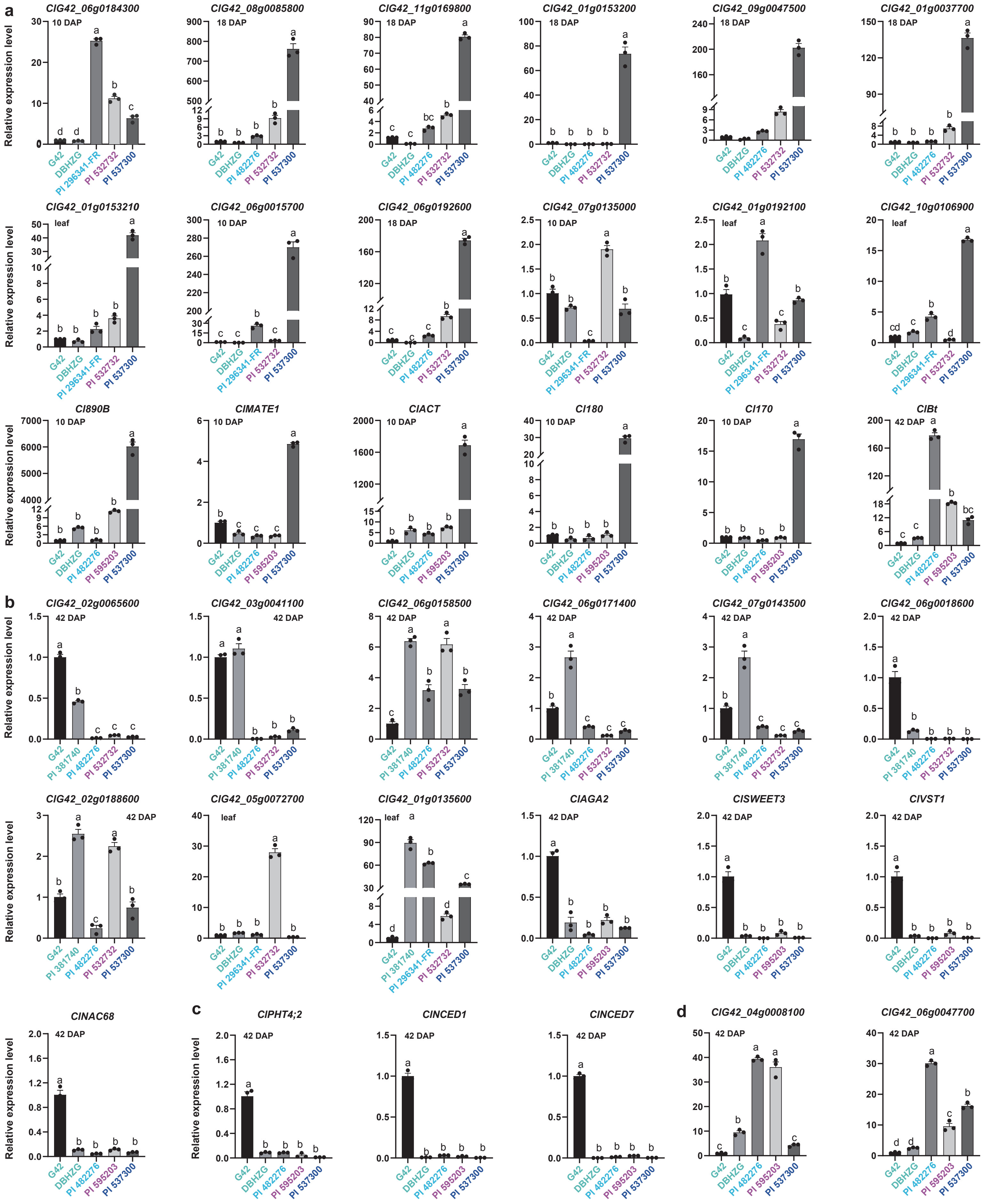

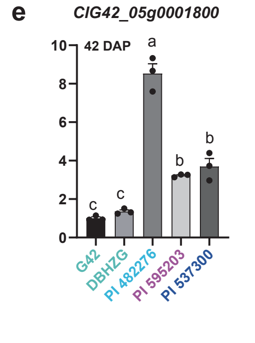

Supplementary Fig. 13 Quantification of relative expressions of SV-genes as related to agronomic traits in fruit flesh by quantitative reverse transcription PCR (qRT-PCR).

(a) Expression of genes related to bitterness in fruit flesh and leaf. (b) Expression of genes related to sugar content in fruit flesh and leaf. (c) Expression of genes related to flesh color in fruit flesh. (d) Expression of genes related to fruit ripening in fruit flesh. (e) Expression of genes related to fruit size in fruit flesh. The fruit stage used for each gene was provided, DAP is abbreviation of days after pollination. Relative expression of different genes was determined by RT-qPCR (n = 3). Data are presented as mean values; means ± s.e. Different lowercase letters indicate a significant difference ($P < 0.05$) based on one-way ANOVA with Tukey's test.

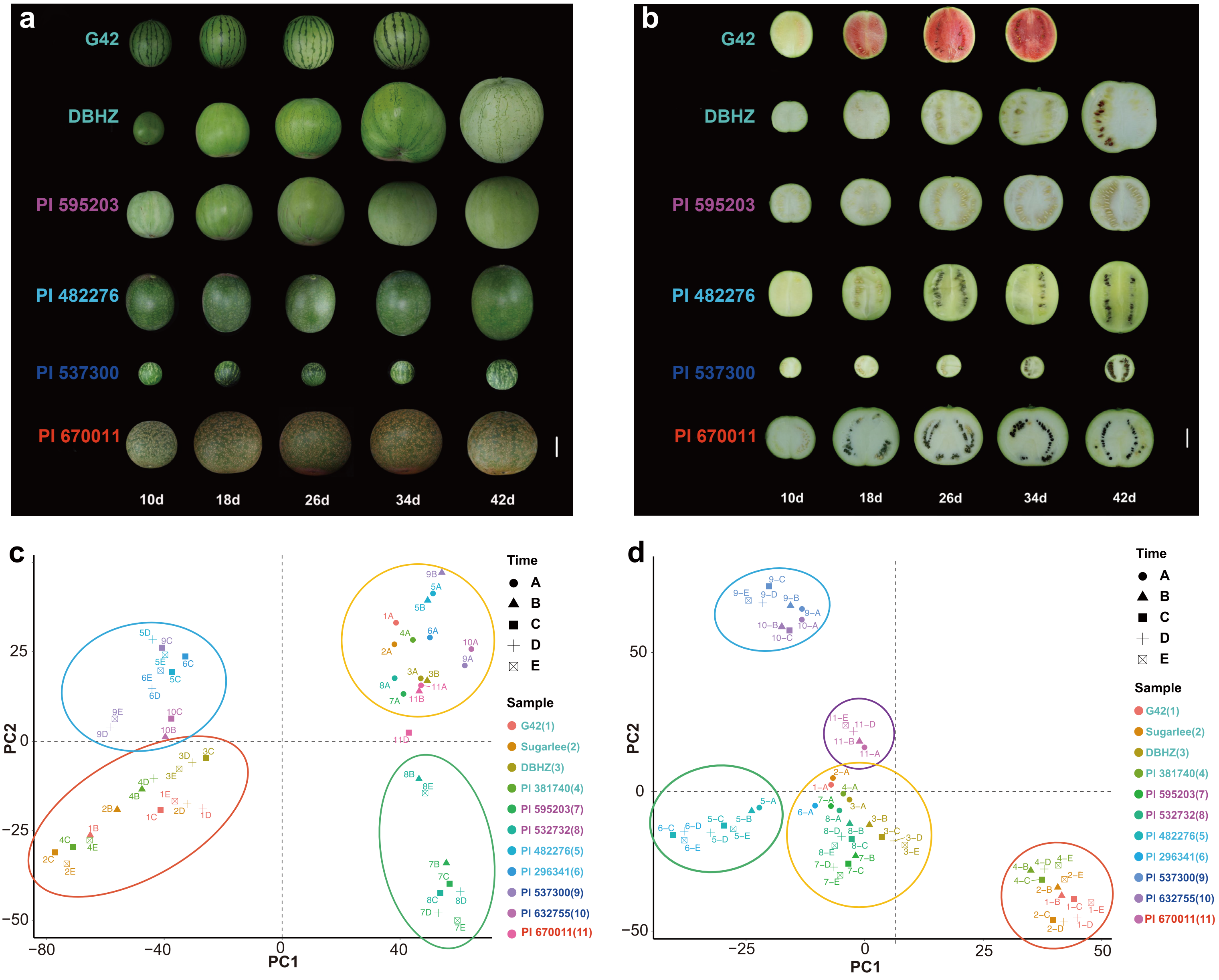

Supplementary Fig. 14 The transcriptomic and metabolomic diversity.

(a-b) Principal Component Analysis (PCA) illustrating the transcriptomic (a) and metabolomic (b) diversity among watermelon accessions across various developmental stages. Five distinct shapes represent the five stages of development, while 11 colors correspond to the 11 distinct accessions. (c-d) Fruit diversity among different watermelon species across five developmental stages. Scale bar corresponds to 5 cm. (c) whole fruit. (d) vertical section of fruit.

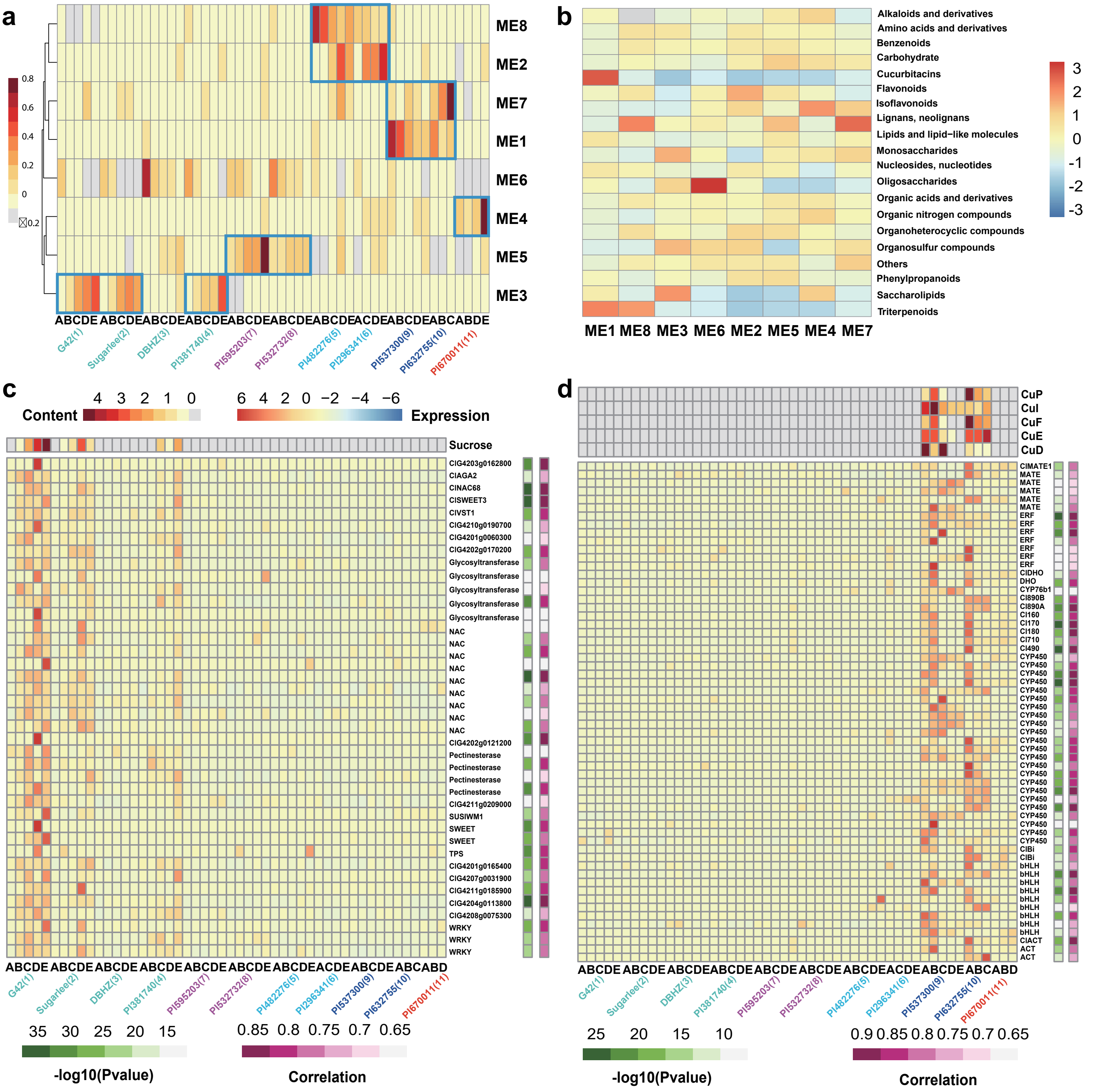

Supplementary Fig. 15 Integrated transcriptomic and metabolomic analysis in 11 watermelon accessions during fruit development.

(a) Eight modules were identified in the metabolomic of WGCNA. Blue boxes denote samples exhibiting high metabolite concentrations within the module. (b) Primary metabolite categories enriched in the eight metabolomic modules. (c-d) Candidate genes implicated in cucurbitacin biosynthesis (c) and sugar metabolism (d) in watermelon. P values were calculated by two-sided Student's t test (c-d).

Additional supplementary figures

unprocessed gels for Supplementary Fig. 8b.

G42_GAP3

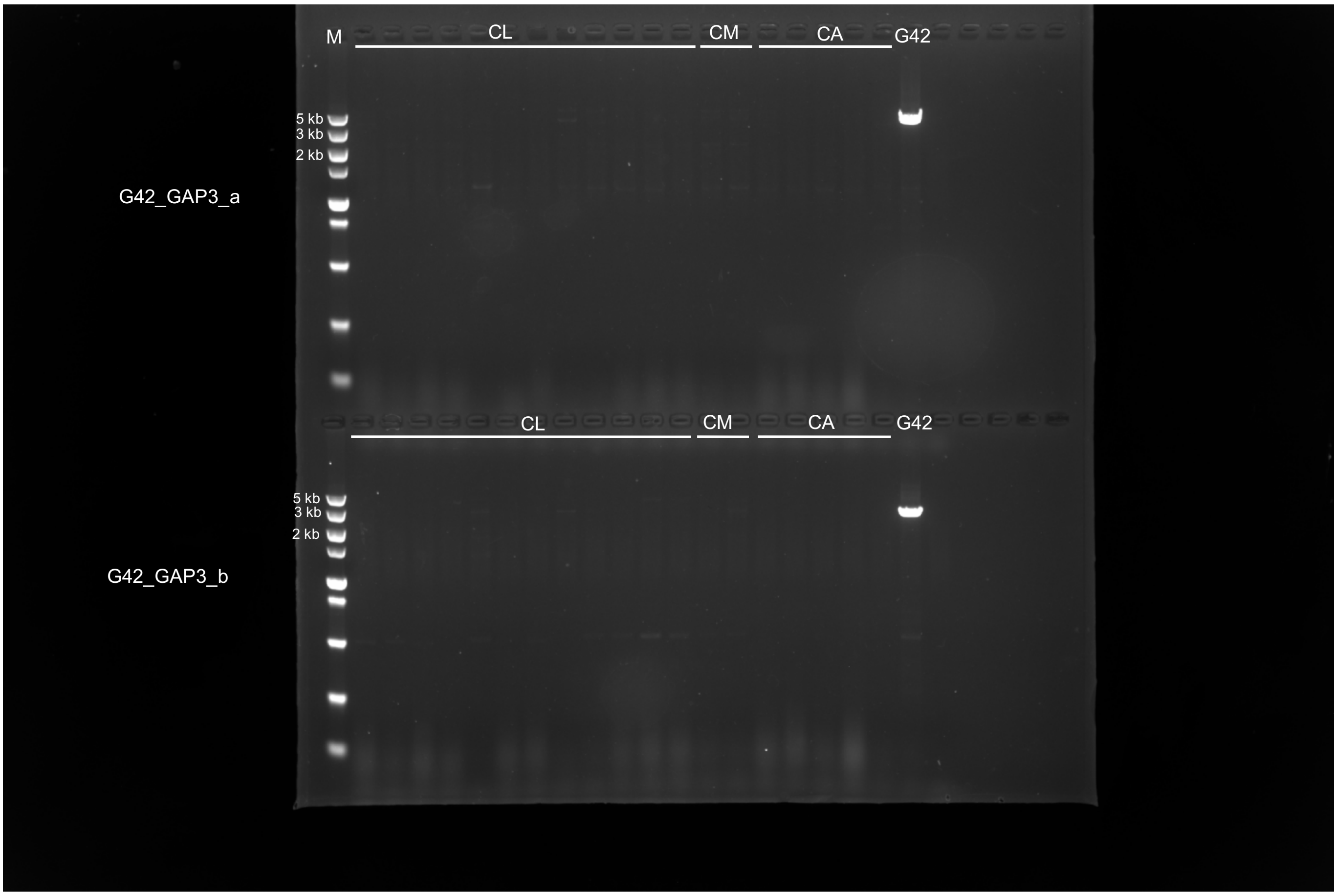

unprocessed gels for Supplementary Fig. 8b.

G42_GAP9

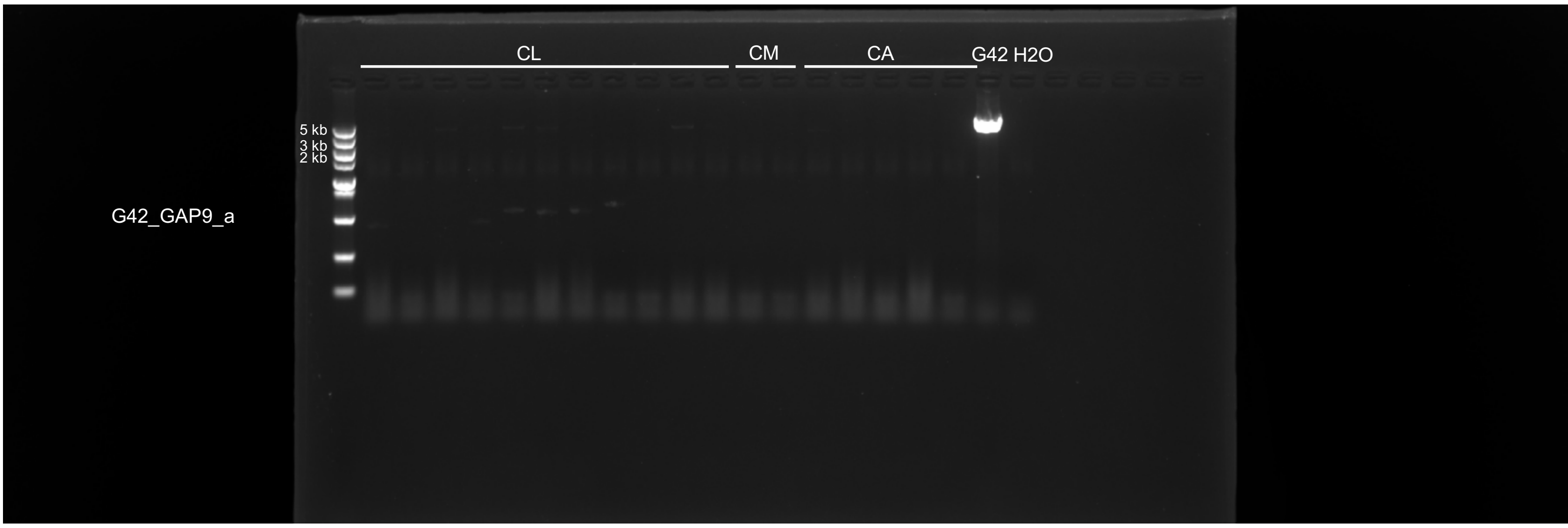

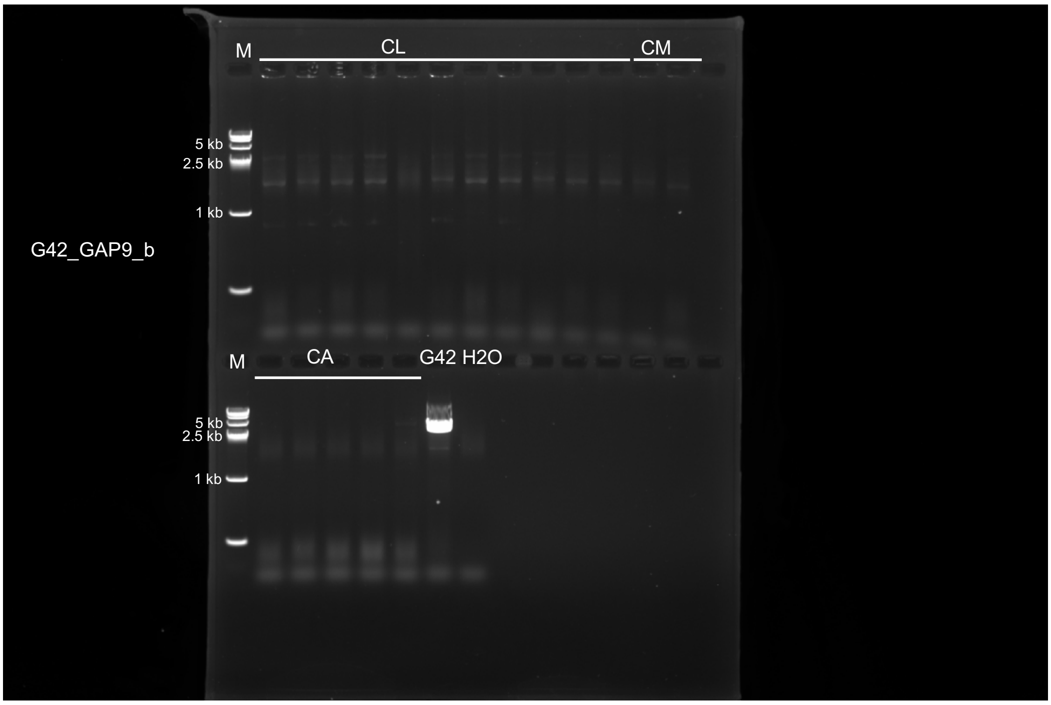

unprocessed gels for Supplementary Fig. 8b.

G42_GAP11

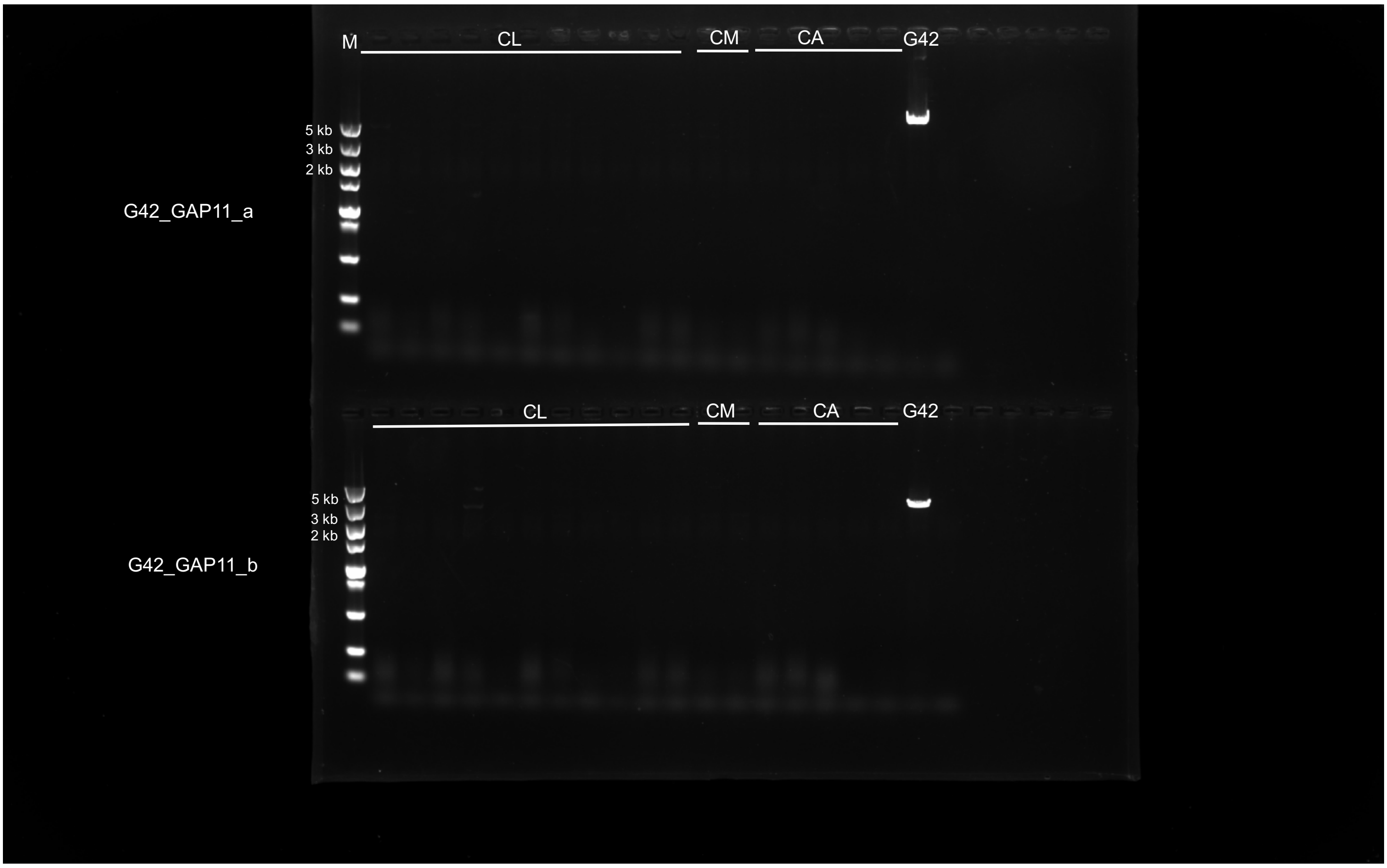

unprocessed gels for Supplementary Fig. 8b.

G42_GAP7

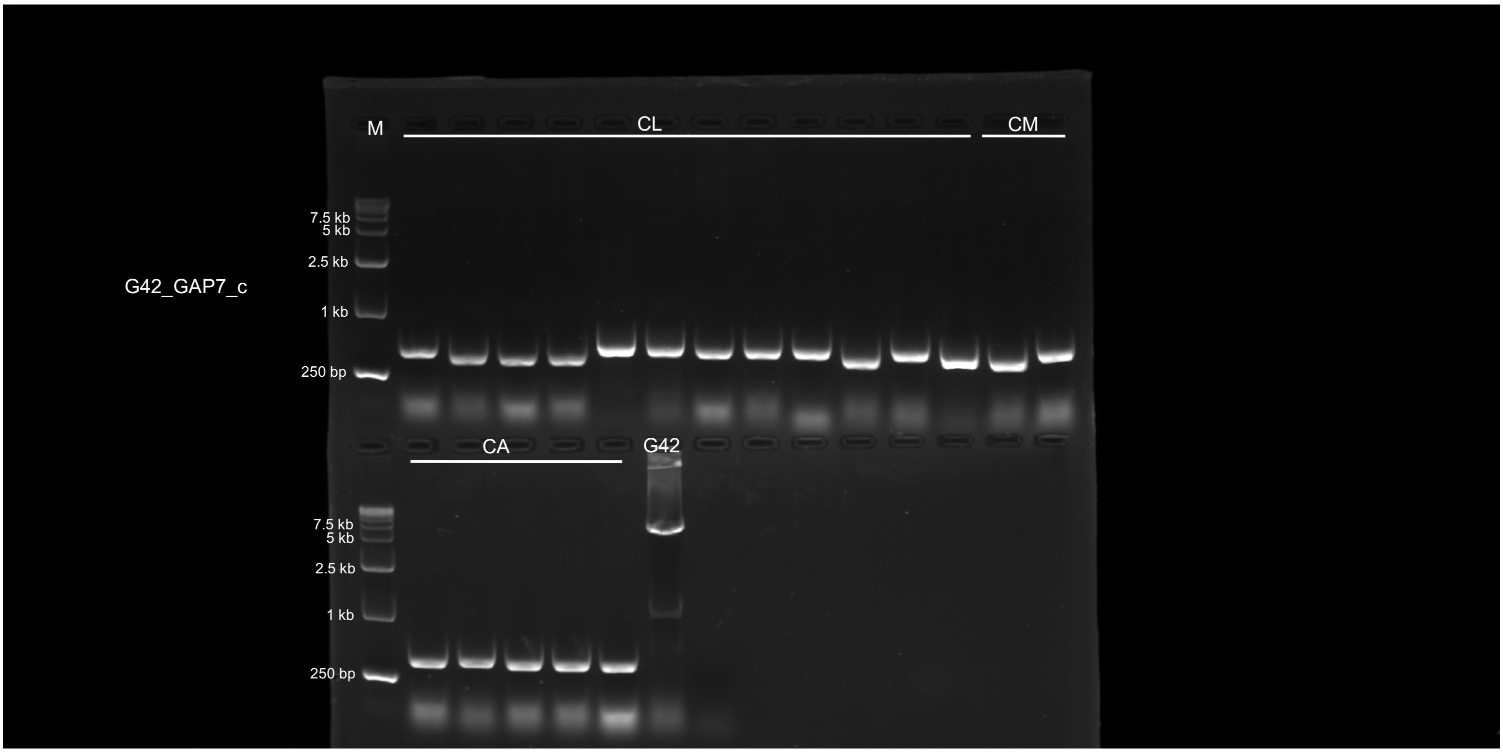

unprocessed gels for Supplementary Fig. 8b.

G42_GAP12

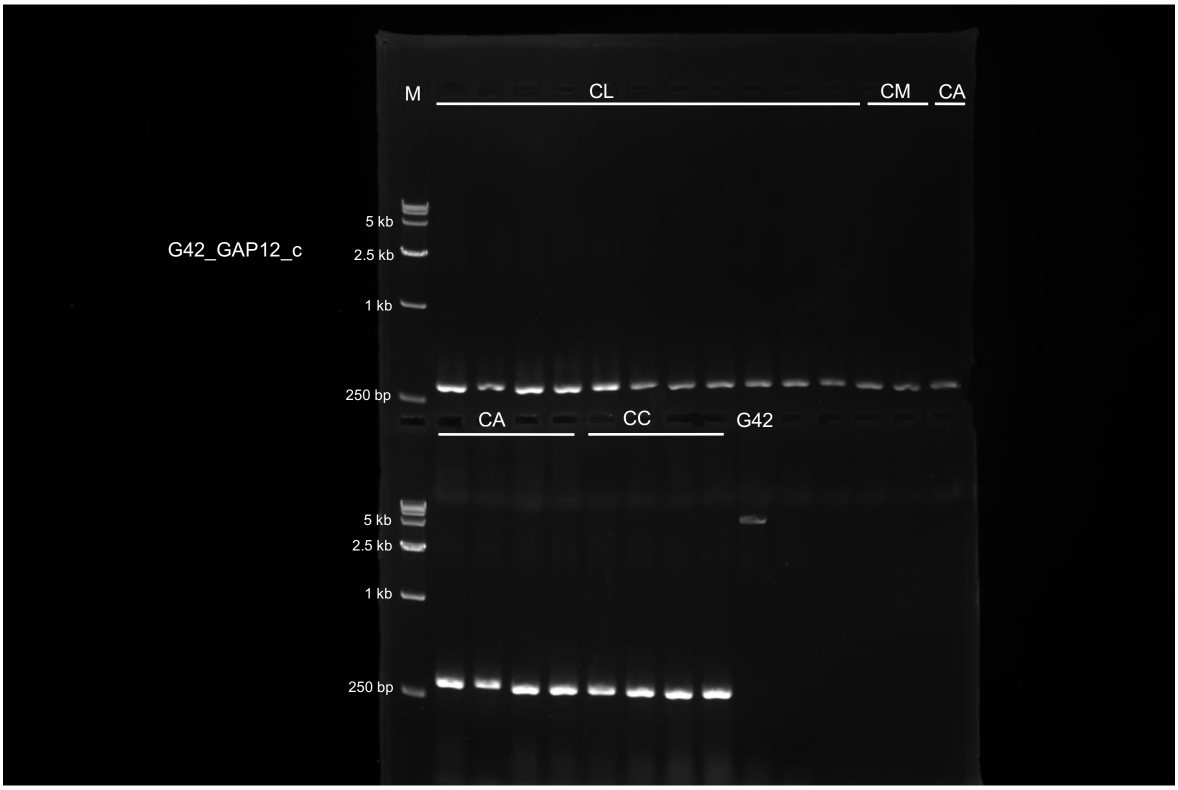

unprocessed gels for Supplementary Fig. 8b.

G42_INS1

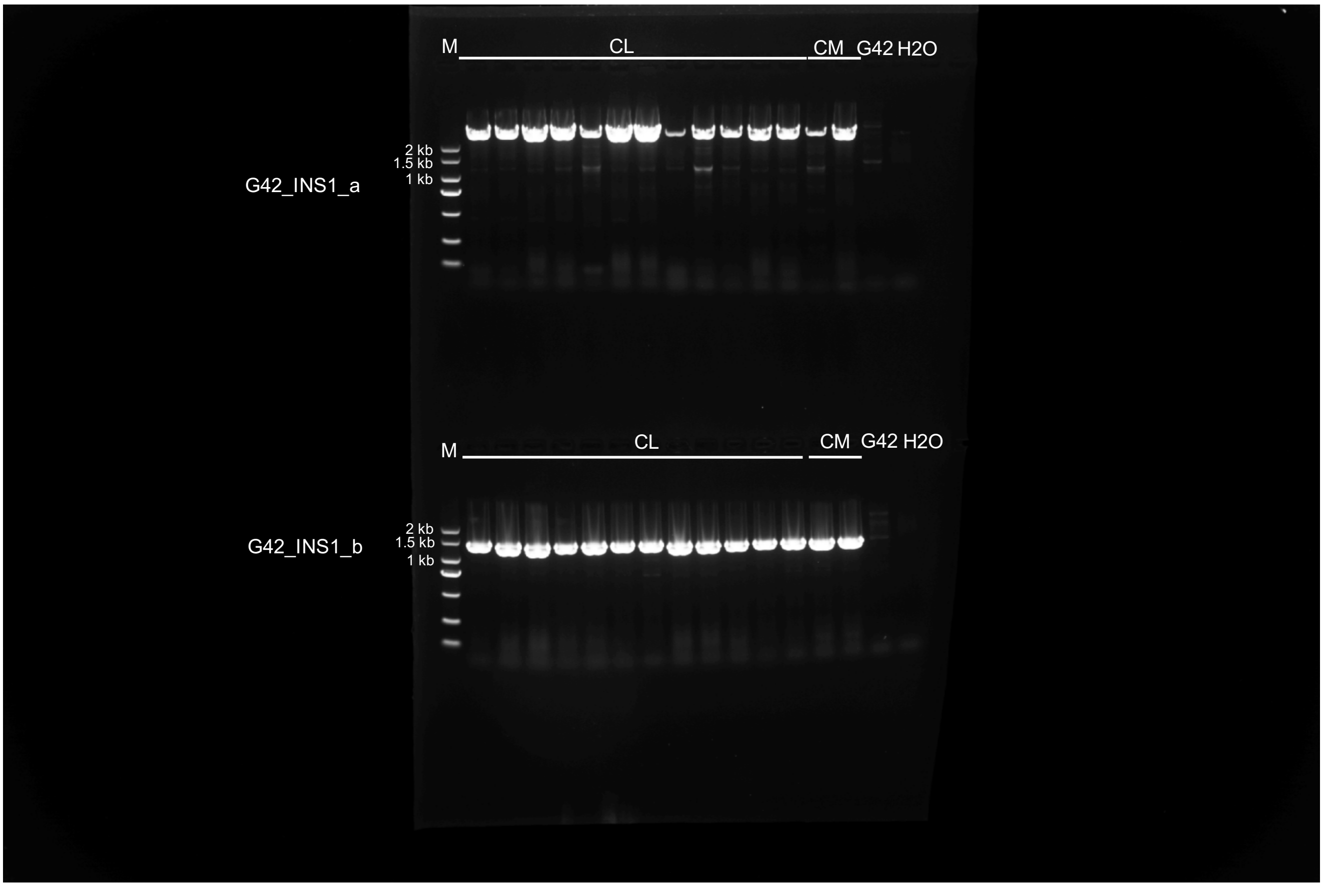

unprocessed gels for Supplementary Fig. 8b.

G42_INS2

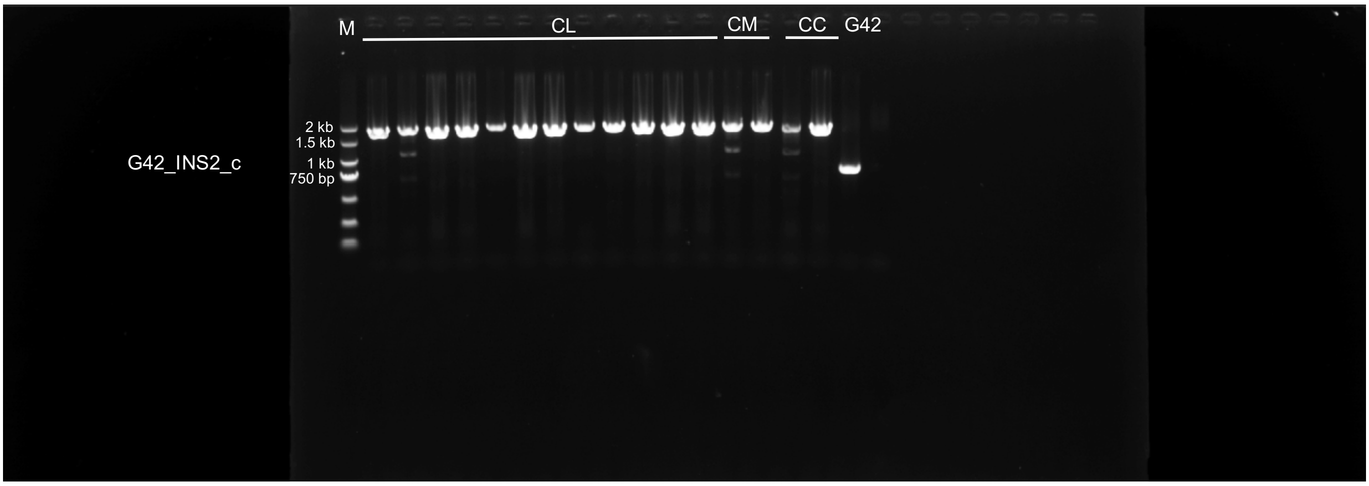

unprocessed gels for Supplementary Fig. 8b.

G42_INS3

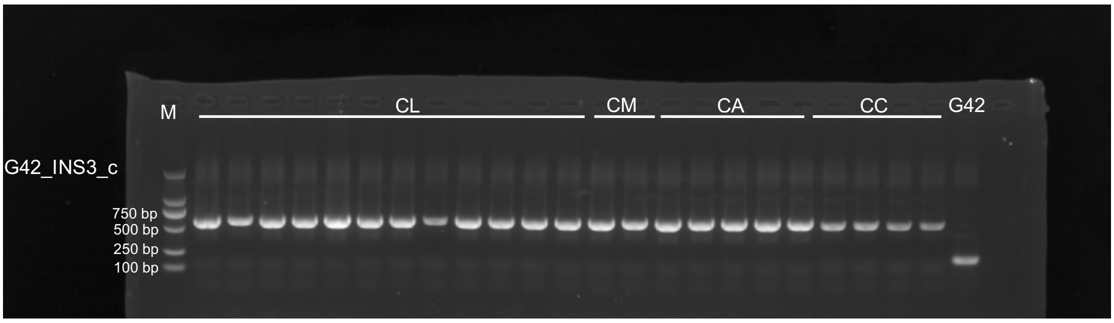

unprocessed gels for Supplementary Fig. 8b.

G42_INS8

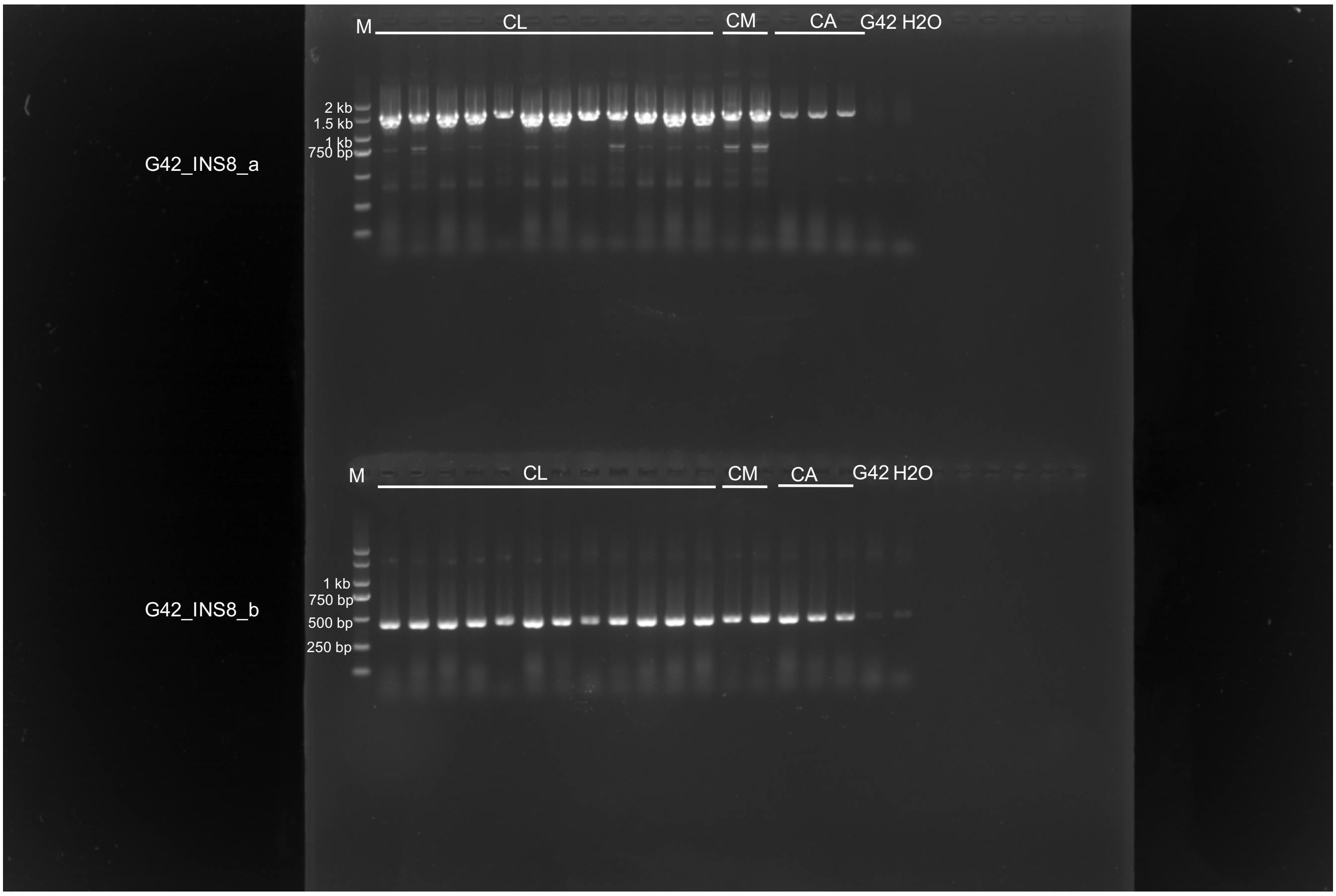

unprocessed gels for Supplementary Fig. 8b.

Wild_GAP1

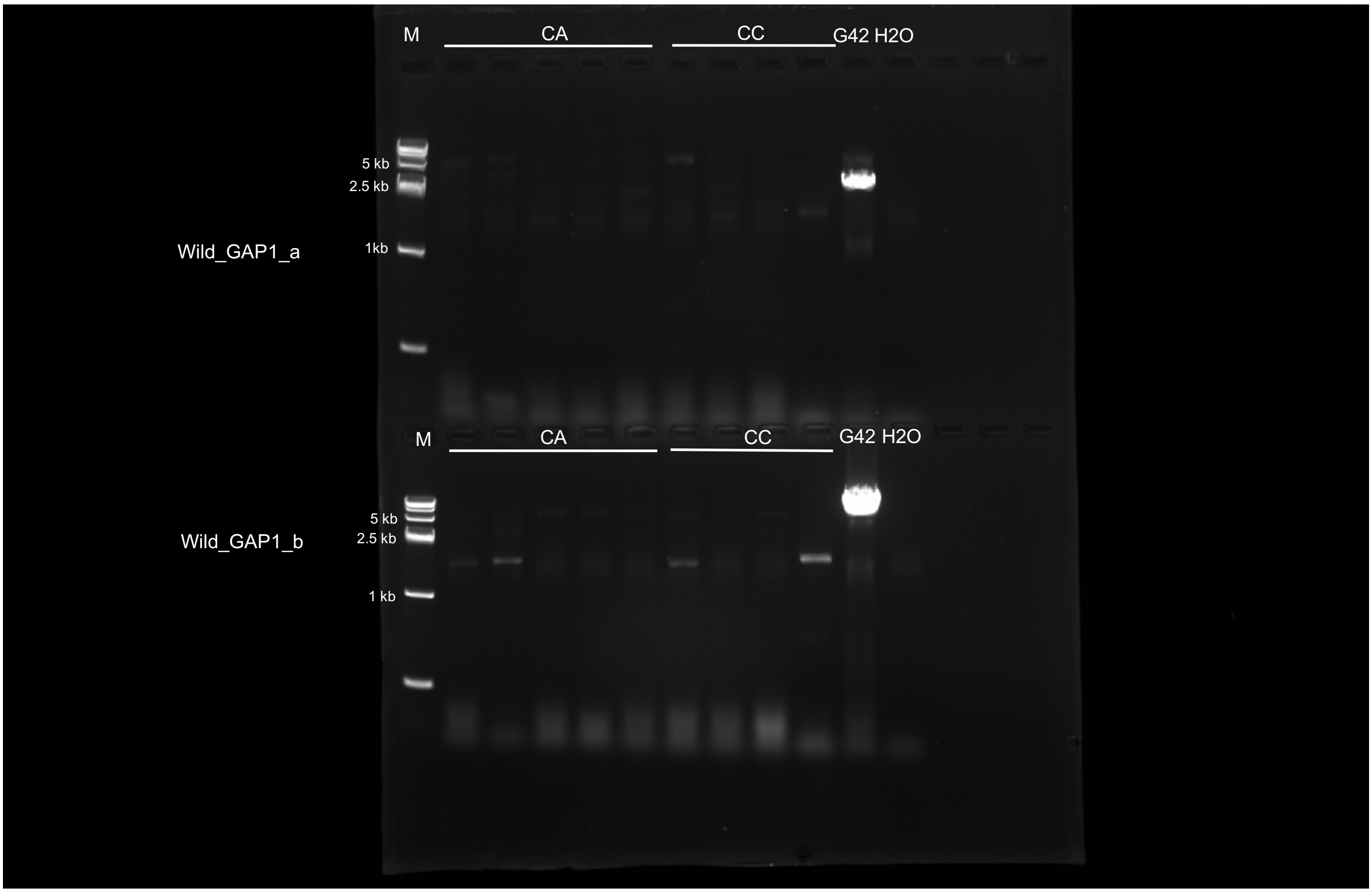

unprocessed gels for Supplementary Fig. 8b.

Wild_GAP3

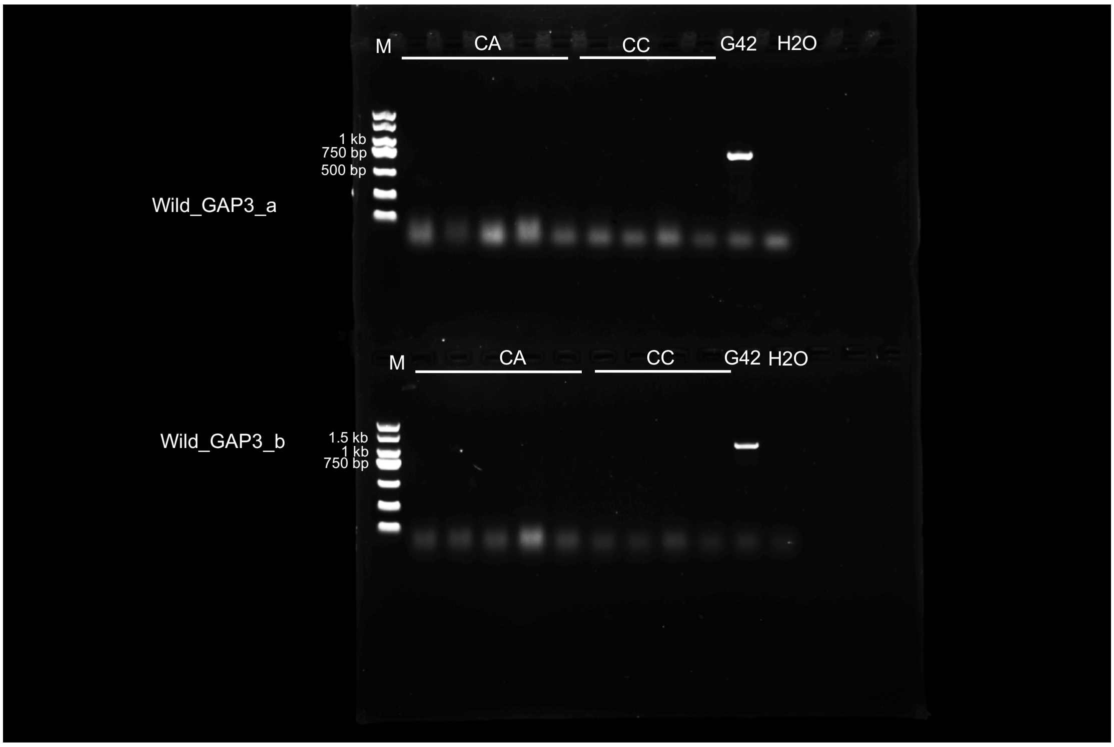

unprocessed gels for Supplementary Fig. 8b.

Wild_GAP9

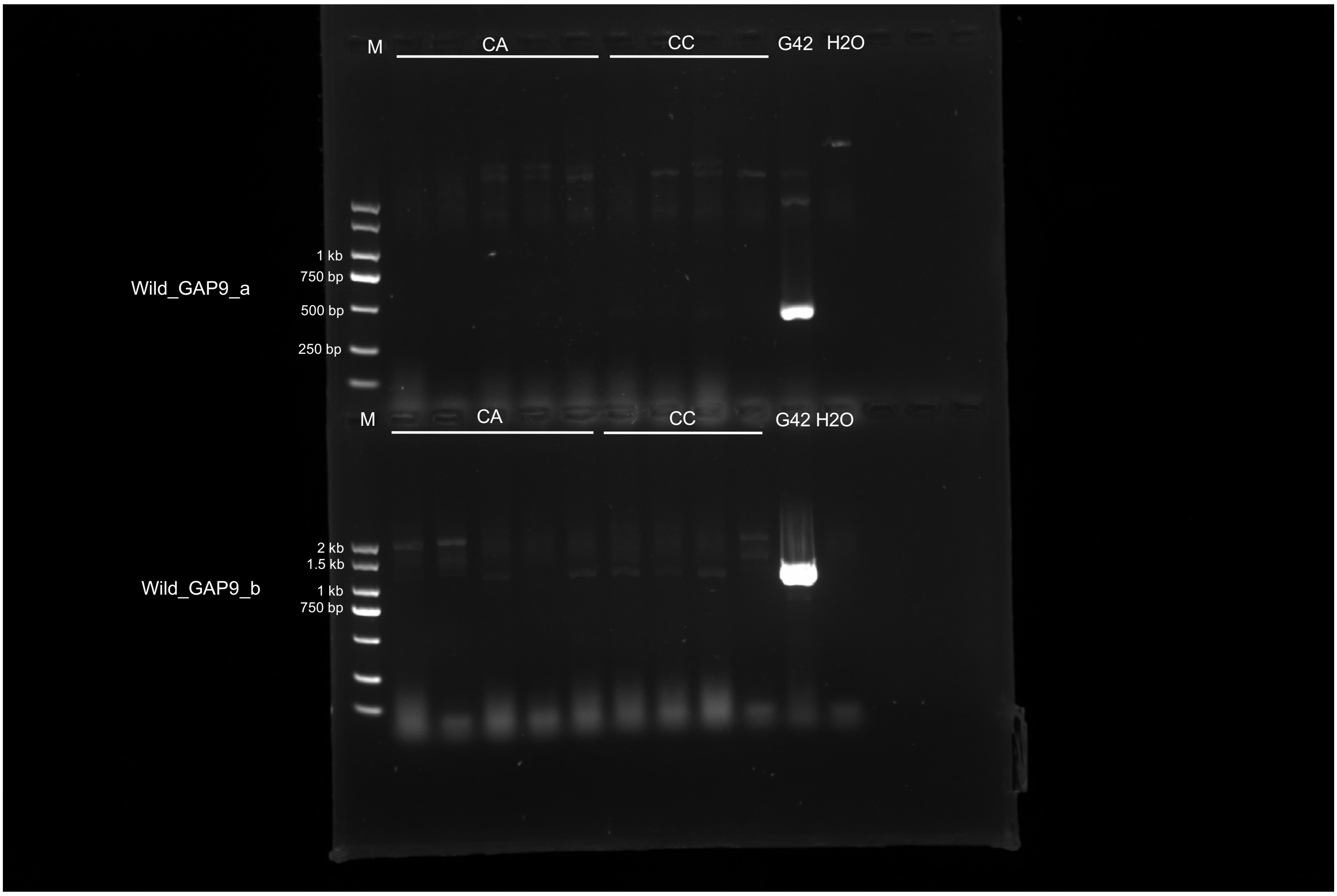

unprocessed gels for Supplementary Fig. 8b.

Wild_GAP16

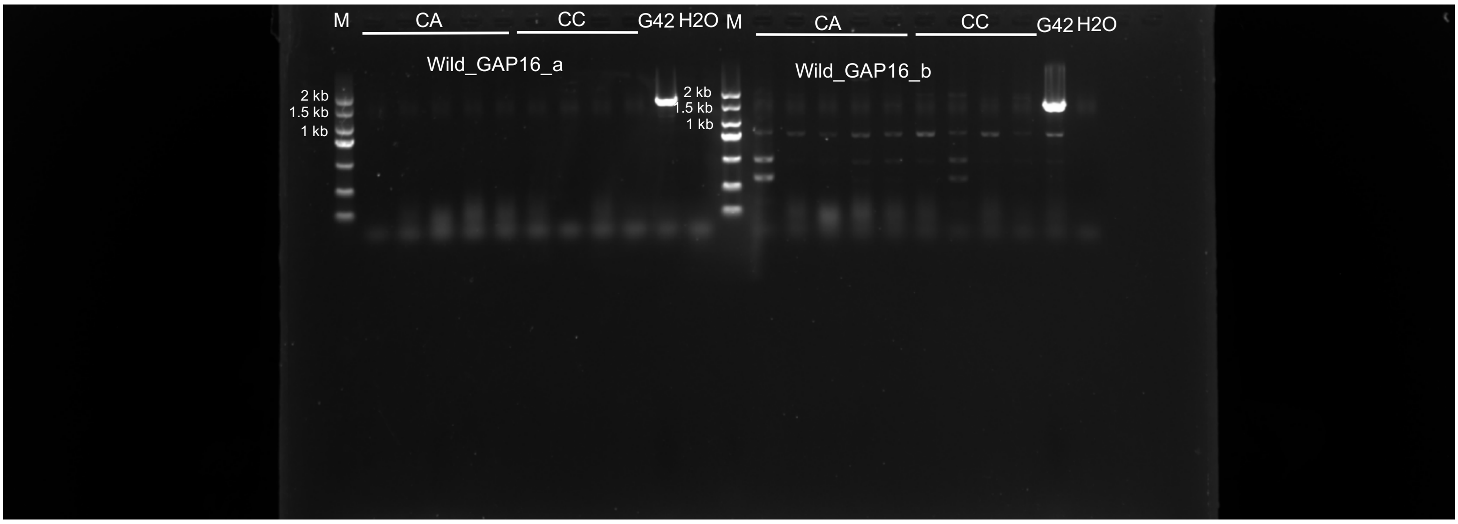

unprocessed gels for Supplementary Fig. 8b.

Wild_INS5

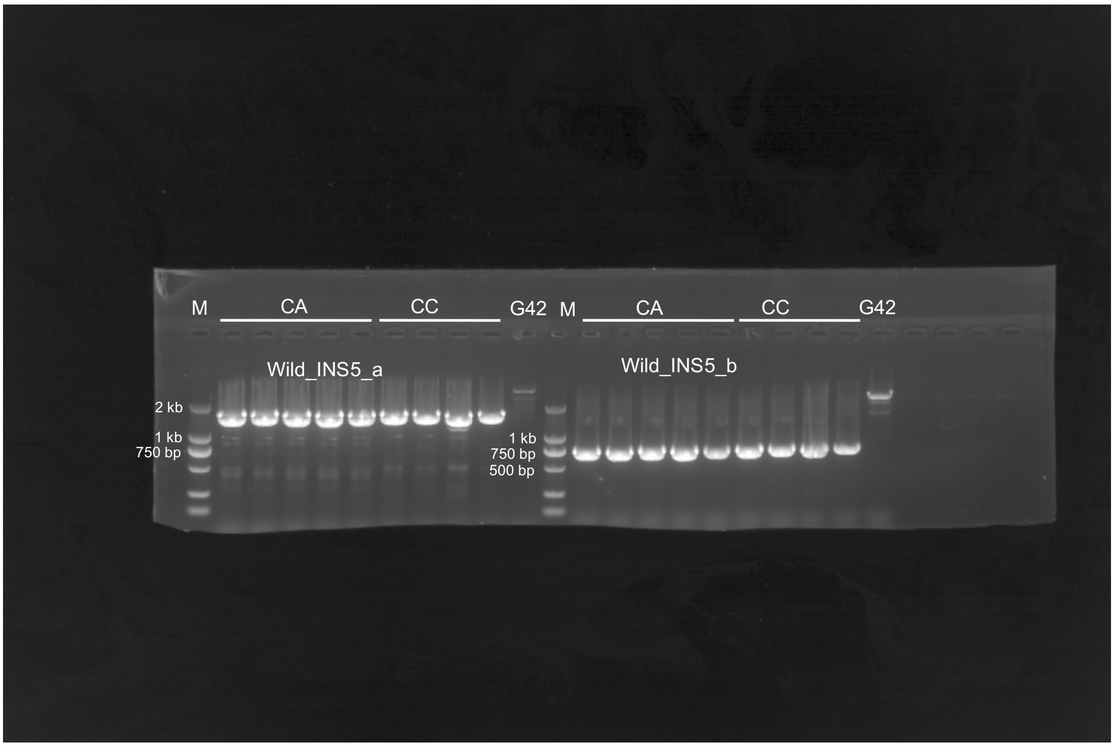

unprocessed gels for Supplementary Fig. 8b.

wild_INS17

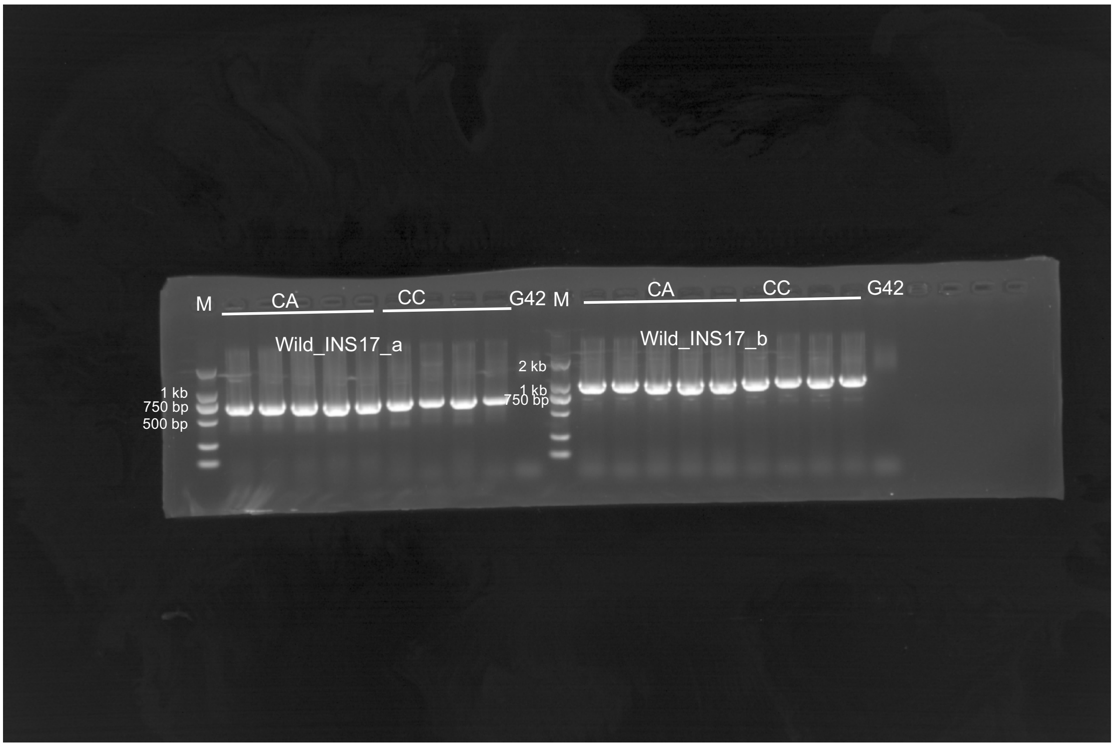

unprocessed gels for Supplementary Fig. 8b.

wild_INS27

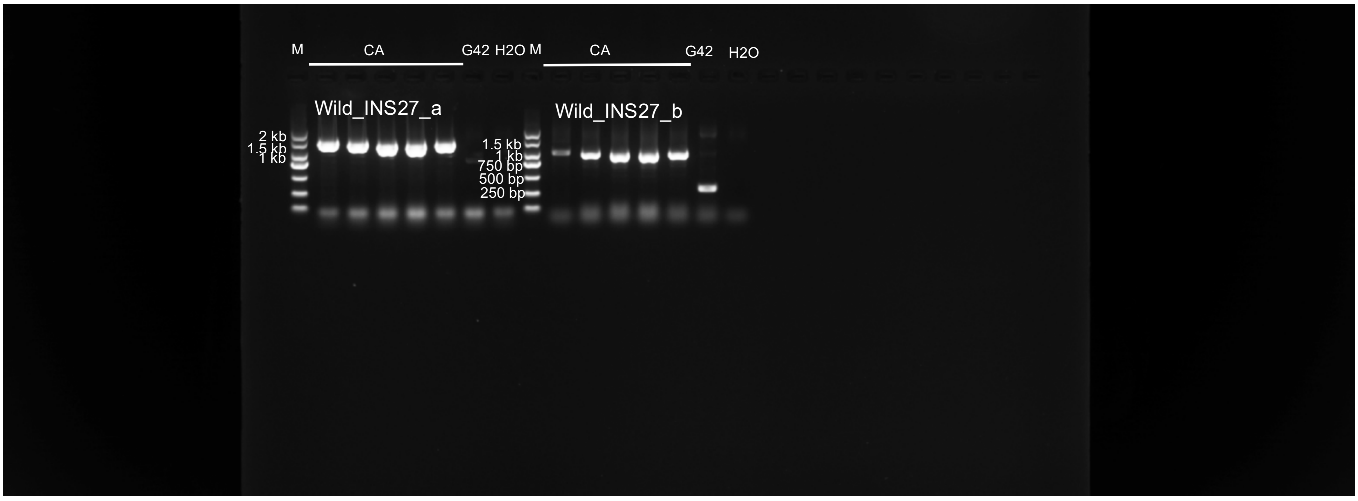

Supplement: Supplementary file 1 — Supplementary Notes 1–6, Supplementary Figs. 1–15 and additional supplementary figures (supporting data for Supplementary Fig. 8b). [file 41588_2024_1823_MOESM1_ESM.pdf]
