# Supplementary figures and images for: Telomere-to-telomere Citrullus super-pangenome provides direction for watermelon breeding
Source: Nat Genet. 2024 Jul 8;56(8):1750–61. doi: 10.1038/s41588-024-01823-6 (PMC11319210; doi:10.1038/s41588-024-01823-6)

# Source data for Figure 4b

## Swap01\_04

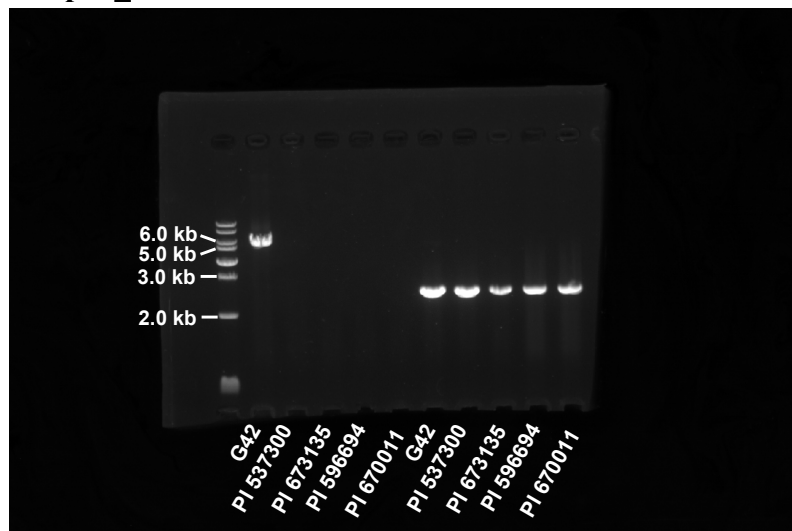

## Swap11\_02

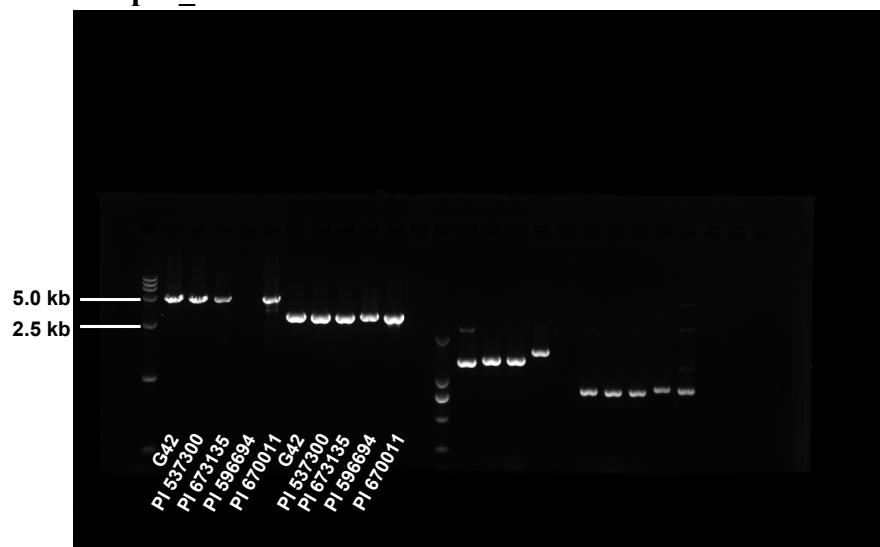

## Swap01\_09

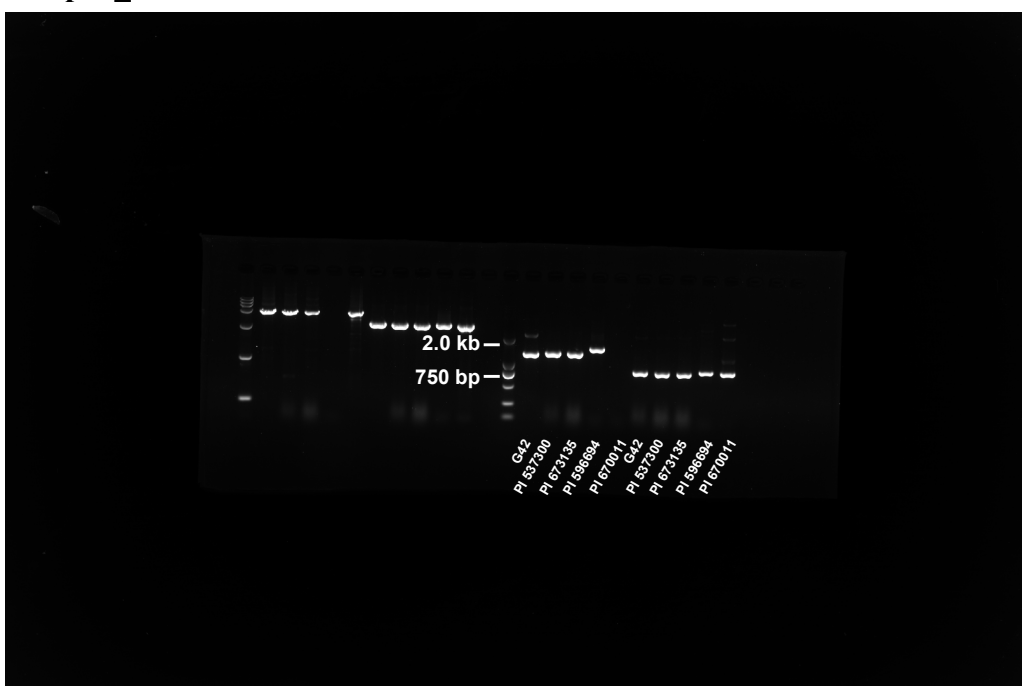

Supplement: Supplementary file 6 — Unprocessed gels. [file 41588_2024_1823_MOESM6_ESM.pdf]
